# Utilizing Causal Network Markers to Identify Tipping Points ahead of Critical Transition

**DOI:** 10.1002/advs.202415732

**Published:** 2025-09-05

**Authors:** Shirui Bian, Zezhou Wang, Siyang Leng, Wei Lin, Jifan Shi

**Affiliations:** ^1^ School of Mathematical Sciences Fudan University Shanghai 200433 China; ^2^ Research Institute of Intelligent Complex Systems Fudan University Shanghai 200433 China; ^3^ Shanghai Center for Mathematical Sciences Fudan University Shanghai 200433 China; ^4^ Institute of AI and Robotics, College of Intelligent Robotics and Advanced Manufacturing Fudan University Shanghai 200433 China; ^5^ Shanghai Artificial Intelligence Laboratory Shanghai 200232 China; ^6^ State Key Laboratory of Medical Neurobiology and MOE Frontiers Center for Brain Science Fudan University Shanghai 200032 China

**Keywords:** causal network marker, early‐warning signal, identification of clinical disease

## Abstract

Early‐warning signals of delicate design are used to predict critical transitions in complex systems, which makes it possible to render the systems far away from the catastrophic state by introducing timely interventions. Traditional signals including the dynamical network biomarker (DNB), based on statistical properties such as variance and autocorrelation of nodal dynamics, overlook directional interactions and thus have limitations in capturing underlying mechanisms and simultaneously sustaining robustness against noise perturbations. This study therefore introduces a framework of causal network markers (CNMs) by incorporating causality indicators, which reflect the directional influence between variables. Actually, to detect and identify the tipping points ahead of critical transition, two markers are designed: the causal network marker from Granger causality (CNM‐GC), for linear causality, and the causal network marker from transfer entropy (CNM‐TE), for non‐linear causality, as well as a functional representation of different causality indicators and a clustering technique to verify the system's dominant group. Through demonstrations using computational benchmark models and real‐world datasets of epileptic seizure, the framework of CNMs shows higher predictive power and accuracy than the traditional DNB. It is believed that, due to the versatility and scalability, the CNMs are suitable for comprehensively evaluating the systems. The most possible direction for application includes the identification of tipping points in clinical disease.

## Graphical abstract

The study proposes a causal network markers (CNMs) framework to identify early‐warning signals preceding critical transitions. It validates CNMs on various computational benchmark models and real‐world datasets, demonstrating higher accuracy and flexibility compared to existing approaches. CNMs also reveal the potential causal patterns in epileptic seizures, thereby deepening the understanding of neurodynamics.

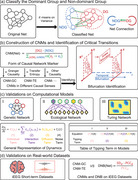

## 1 Introduction

When numerous similar entities at micro‐level engage in interactions among themselves and with their environment, spontaneous and often unexpected outcomes emerge at spatiotemporal macro‐level. This phenomenon, known as emergence, is a hallmark of various complex systems.^[^ ^1^ ^]^ Emergence is frequently linked to collective behavior, wherein many living systems demonstrate critical transition.^[^ ^2^, ^3^, ^4^ ^]^ At these tipping points, systems undergo transitions from normal states to catastrophic states. As a result, extensive investigations have been dedicated to designing early‐warning signal and thus predicting the occurrence of such critical transitions between different states within these systems.^[^ ^5^, ^6^ ^]^ Early‐warning signals of delicate design are essential for predicting critical transitions in complex dynamical systems, which makes it possible to conduct timely interventions, prevent catastrophic events and mitigate negative impacts. Some traditional methods, e.g., the dynamical network biomarker (DNB), rely on statistical quantities such as variance, autocorrelation, and dimension reduction, which offers valuable insights into the system's state.^[^ ^3^, ^6^, ^7^, ^8^, ^9^ ^]^ However, such methods have difficulties in capturing the directional interactions and underlying mechanisms that drive the dynamics of complex systems.

Causality indicates a directional influence between a pair of variables and is a fundamental form of interactions within complex systems. Researchers have developed various sophisticated causality indicators to characterize the relationships between nodes in complex networks, such as the Granger causality (GC), the transfer entropy (TE), and the embedding entropy (EE).^[^ ^10^, ^11^, ^12^ ^]^ These indicators, due to their broad applicability and diversity, hold significant potential for enhancing our understanding of system dynamics in different senses. For instance, approaches based on Takens' embedding theorem effectively characterize the relationship between non‐linearly coupled nodes and solve a critical causal inference problem in terms of non‐separability.^[^ ^11^, ^13^ ^]^ Inspired by these advances, incorporating causality indicators into the framework of early‐warning signals may provide a more robust and informative approach for identifying critical points and predicting system bifurcations.

In this study, we proposed a framework of causal network markers (CNMs) to detect typical critical bifurcations in the dynamical evolution of complex systems. Our framework begins with the *K*‐means clustering method based on data variance, categorizing nodes into dominant group (DG) and non‐dominant group (NDG). More importantly, our theoretical analysis shows that as the system nears a tipping point, the unidirectional GC from DG nodes to NDG nodes vanishes. Leveraging this property, we construct CNMs to indicate the extent to which the dynamics approaches a tipping point. Specifically, we select GC and TE under the framework of CNM, viz., CNM‐GC, which quantifies “linear causality”, and CNM‐TE, which quantifies “non‐linear causality”.

To validate the efficacy of CNM‐GC and CNM‐TE, we applied them to both computational benchmark models and real‐world datasets. The benchmark models include a five‐gene network, an ecological network, and a Turing diffusion interaction network, each representing different critical phenomena. Our results demonstrate that CNMs have significant predictive power for both temporal and spatial bifurcation models. In addition, we tested our markers using the epileptic seizure dataset, intracranial electroencephalography (iEEG), where the combination of the two markers showed high predictive accuracy. It is worth noting that due to the complexity of epilepsy dynamics, combining CNM‐GC and CNM‐TE information could further identify the types of causal state that trigger the tipping point. By integrating multiple causality indicators, CNMs offer a comprehensive identification of system dynamics, making them suitable for applications in clinical disease detection and early‐warning.

## 2 Results and Discussions

### 2.1 Vanishment of Causality Near the Tipping Point

Here, we illustrate that when the system's dynamics approaches a tipping point, the causality from the DG nodes to the NDG nodes vanishes, in the sense of GC and TE. Generally, a discrete dynamical system with multidimensional parameter P is governed by

(1) $$\begin{array}{c} Z^{t+1}=f\left(Z^{t};P\right) \end{array}$$

where $Z^{t}={\left(z_{1}^{t},z_{2}^{t},\cdots ,z_{n}^{t}\right)}^{⊤}$ is a vector with *n* components, and f is a continuously differentiable function with a non‐trivial fixed point $\bar{Z}$, i.e., $f\left(\bar{Z}\right)=\bar{Z}$. Assume there exists a tipping point at parameter $P_{c}$, where the system undergoes a codimension‐one bifurcation. Linearization of the system around $\bar{Z}$ yields

(2) $$\begin{array}{c} X^{t+1}=AX^{t}+\Gamma^{t} \end{array}$$

where $X^{t}=Z^{t}-\bar{Z}$,

(3) $$\begin{array}{c} A={\left.\frac{\partial f(Z;P)}{\partial Z}\right|}_{Z=\bar{Z}} \end{array}$$

is the Jacobian matrix of f at $\bar{Z}$, and $\Gamma^{t}={\left(\Gamma_{1}^{t},\cdots ,\Gamma_{n}^{t}\right)}^{⊤}$ contains all remaining high‐order terms. To directly display our conclusion, we write down a typical situation where A can be diagonalized in $R^{n\times n}$: $A=S\Lambda S^{-1}$ with $S,\;\Lambda \in R^{n\times n}$. Set $Y^{t}=S^{-1}X^{t}$ and we can obtain

(4) $$\begin{array}{cc} & SY^{t+1}=ASY^{t}+\Gamma^{t}=\left(S\Lambda S^{-1}\right)SY^{t}+\Gamma^{t},\\ & Y^{t+1}=\Lambda Y^{t}+S^{-1}\Gamma^{t} \end{array}$$

where $Y^{t}={\left(y_{1}^{t},y_{2}^{t},\cdots ,y_{n}^{t}\right)}^{⊤}$ satisfies a diagonal dynamic. In the following discussion, we assume *y*_1_ to be the only one “dominant variable” in this diagonal dynamic. That is, when P goes to $P_{c}$, the variance of *y*_1_ tends to infinity, while the Pearson correlation coefficients (PCC) between *y*_1_ and other variables tend to 0.^[^ ^6^ ^]^ Now we calculate the first‐order GC between any two variables *x*_*i*_ and *x*_*j*_ in the original phase space.^[^ ^10^ ^]^

For $x_{i}^{t}=s_{i1}y_{1}^{t}+\cdots +s_{in}y_{n}^{t}$ and $x_{j}^{t}=s_{j1}y_{1}^{t}+\cdots +s_{jn}y_{n}^{t}$, there are *H*_0_‐model and *H*_1_‐model for the causality from *x*_*j*_ to *x*_*i*_:

(5) $$\left\{\begin{array}{c} \begin{array}{c} H_{0}:\;\;x_{i}^{t+1}=ax_{i}^{t}+\xi_{1}, \end{array}\\ \begin{array}{c} H_{1}:\;\;x_{i}^{t+1}=bx_{i}^{t}+cx_{j}^{t}+\xi_{2} \end{array} \end{array}\right.$$

Based on Equation (5), we prove that when *s*_*i*1_ = 0 and *s*_*j*1_ ≠ 0, i.e., the *j*‐th variable is related to the dominant variable $y_{1}^{t}$ while the *i*‐th not, $E\left[\xi_{2}^{2}\right]\to E\left[\xi_{1}^{2}\right]$ holds as the variance of *y*_1_ tends to infinity. Thus, the Granger causality strength from $x_{j}^{t}$ to $x_{i}^{t}$, ${\text{GC}}_{x_{j}\to x_{i}}=\log\frac{E[\xi_{1}^{2}]}{E[\xi_{2}^{2}]}$, tends to 0. Besides, we discuss the other three situations (see detailed proof in Supporting Information). The conclusions for the four cases are summarized as

a) *s*_*i*1_ = 0, *s*_*j*1_ ≠ 0, and ${\text{GC}}_{x_{j}\to x_{i}}$ tends to 0;
b) *s*_*i*1_ ≠ 0, *s*_*j*1_ = 0, and ${\text{GC}}_{x_{j}\to x_{i}}$ is bounded;
c) *s*_*i*1_ ≠ 0, *s*_*j*1_ ≠ 0, and ${\text{GC}}_{x_{j}\to x_{i}}$ is bounded;
d) *s*_*i*1_ = 0, *s*_*j*1_ = 0, and ${\text{GC}}_{x_{j}\to x_{i}}$ is invariant.

The results indicate that in sense of GC, when the system attains a tipping point, the causality from the variable corresponding to the dominant diagonalized variables (i.e., the variables in DG), to those in NDG tends to be 0, while the causality from the variables in NDG to those in DG changes within a bounded number. Meanwhile, the causality between variables in NDG does not change. Furthermore, the causality vanishing also holds for TE. This is because when the stochastic system evolves nearby an attractor representing a stable biophysical state, Kurtz's central limit theorem and corresponding theoretical improvement have shown that the evolution is approximately Gaussian.^[^ ^14^, ^15^, ^16^, ^17^, ^18^, ^56^ ^]^ Given that TE reduces to GC under Gaussian assumptions^[^ ^57^ ^]^, we infer that the properties observed for GC extend to TE, as will be demonstrated in our subsequent results. Our additional experiments reveal that this phenomenon also manifests in EE within a simplified system, suggesting that the “causality vanishing” between DG and NDG represents a robust and generalizable property (see Supporting Information).

### 2.2 A Framework of Causal Network Markers for Tipping Point Identification

In the previous section, it is shown that the causality strength of GC rapidly changes when the system evolves in the vicinity of the tipping point. Empirically, GC reflects causality in a “linear sense”. Naturally, we can extend the idea to a broader sense of causality, such as TE and other causality indicators.

TE is a non‐parametric statistic that measures the amount of directional (time asymmetric) information transfer between two random processes. In the general sense, the TE is designed to reveal causality in the “non‐linear sense”. Specifically, the calculation of first‐order TE is given in the following form: TE_*X* → *Y*_ = *H*(*Y*_*t* + 1_∣*Y*_*t*_) − *H*(*Y*_*t* + 1_∣*Y*_*t*_, *X*_*t*_), where *H*(· | ·) represents the conditional entropy. Therefore, in this work, we not only use GC, but also introduce TE.

Based on the previous proof and analysis of the “causal vanishing” property, we propose a network marker framework with GC or TE to detect critical transitions, which can be easily extended to other types of causality (such as EE), in principle. First, we divide nodes into DG and NDG via the *K*‐means algorithm, where the clusters are obtained by the average variance of nodes during the given period (see pseudocode in Supporting Information). After that, our marker is calculated based on the clusters as follows:

(6) $$\text{CNM(Net)}:=\frac{\left|\text{DG}|\cdot |\text{NDG}\right|}{\sum_{j\in \text{DG},i\in \text{NDG}}\text{cs}\left(x_{j}\to x_{i}\right)}$$

where | · | represents the number of the elements in a set, and cs(·) is the causality strength in the sense of GC, TE, or other types of causality indicators.

**Figure 1:**
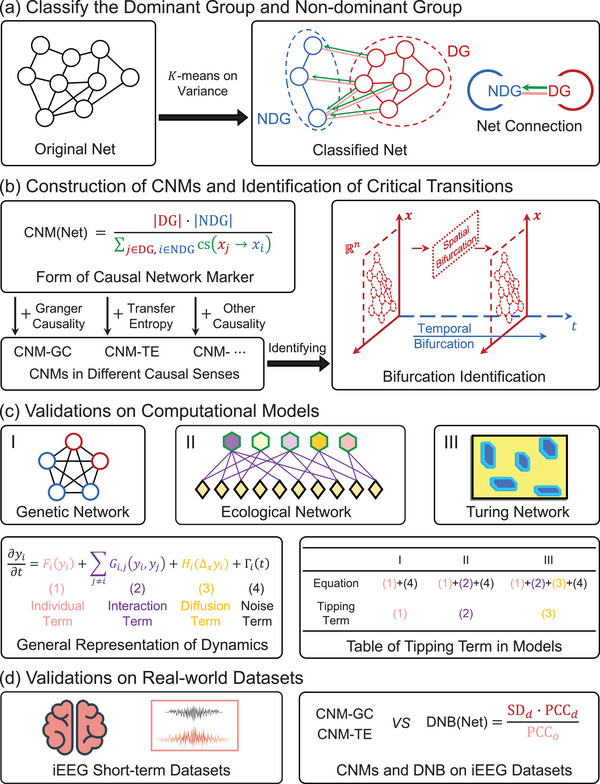
A sketch depicting the main procedure for calculating the CNMs. a) Verifying the DG and the NDG through the *K*‐means algorithm. b) Calculating the CNMs with different causality indicators, such as GC and TE. c) CNMs' validation on detecting the general bifurcation phenomena on benchmark models. d) CNMs' validation on the iEEG seizure dataset, exhibits a more scalable and flexible approach to detecting the tipping point of real‐world datasets.

Through the CNM, all of the elements approaching 0 in the set of {*x*_*j*_ ∈ DG, *x*_*i*_ ∈ NDG | cs(*x*_*j*_ → *x*_*i*_) → 0}, thus the marker blows up. Therefore, we use the significant increase in the marker as an effective early‐warning signal for tipping points. In **Figure** **1**, we sketch the major procedure for the CNMs framework.

### 2.3 Validations on Computational Benchmark Models

Here, we apply the CNMs framework combined with two types of causality indicators to computational benchmark models. From the complex biological networks' perspective, the bifurcation of system evolution usually has different internal causes, led by different terms in the dynamical equations.^[^ ^19^, ^20^ ^]^ Specifically, the representation of complex dynamics with *n* nodes is governed as [21]:

(7) $$\begin{array}{cc} \frac{\partial y_{i}}{\partial t}= & F_{i}\left(y_{i}\right)+\sum_{j\neq i}G_{i,j}\left(y_{i},y_{j}\right)\\ & +H_{i}\left(\Delta_{x}y_{i}\right)+\Gamma_{i}\left(t\right),\;i=1,2,\cdots ,n \end{array}$$

where $y_{i}=y_{i}\left(t;x\right)$ is a temporal‐spatial variable, *F*_*i*_ is the individual evolving dynamics, *G*_*i*, *j*_ is the interaction term between nodes, *H*_*i*_ is the spatial diffusion term for the spatial states x, and Γ_*i*_ is Gaussian white noise with:

(8) $$\begin{array}{c} \langle \Gamma_{i}\left(t\right)\rangle =0,\;\langle \Gamma_{i}\left(t\right)\Gamma_{j}\left(t^{\prime }\right)\rangle =2D\delta_{ij}\delta \left(t-t^{\prime }\right) \end{array}$$

To test the effectiveness of our framework under different types of bifurcation phenomena, three typical biological networks are selected from the application, namely, the genetic networks, the ecological networks, and the Turing networks. Numerical experiments show that CNM‐GC and CNM‐TE can identify these simple network models with different types of bifurcation phenomena effectively.

#### 2.3.1 Five‐Gene Genetic Network

The five‐gene genetic network is a regulatory network and the system is governed by 5‐dimensional Langevin equations:^[^ ^6^, ^22^, ^23^ ^]^

(9) $$\left\{\begin{array}{c} \begin{array}{cc} \frac{dz_{1}\left(t\right)}{dt}= & \left(90|P|-1236\right)+\frac{240-120|P|}{1+z_{3}\left(t\right)}\\ & +\frac{1488z_{4}\left(t\right)}{1+z_{4}\left(t\right)}-30\left|P\right|z_{1}\left(t\right)+\Gamma_{1}\left(t\right), \end{array}\\ \begin{array}{cc} \frac{dz_{2}\left(t\right)}{dt}= & \left(75|P|-150\right)+\frac{60-30|P|}{4z_{1}\left(t\right)-2}\\ & +\frac{\left(240-120|P|\right)z_{3}\left(t\right)}{1+z_{3}\left(t\right)}-60z_{2}\left(t\right)+\Gamma_{2}\left(t\right), \end{array}\\ \begin{array}{c} \frac{dz_{3}\left(t\right)}{dt}=-1056+\frac{1488z_{4}\left(t\right)}{1+z_{4}\left(t\right)}-60z_{3}\left(t\right)+\Gamma_{3}\left(t\right), \end{array}\\ \begin{array}{c} \frac{dz_{4}\left(t\right)}{dt}=-600+\frac{1350z_{5}\left(t\right)}{1+z_{5}\left(t\right)}-100z_{4}\left(t\right)+\Gamma_{4}\left(t\right), \end{array}\\ \begin{array}{cc} \frac{dz_{5}\left(t\right)}{dt}= & 108+\frac{160}{1+z_{1}\left(t\right)}+\frac{40}{1+z_{2}\left(t\right)}\\ & +\frac{1488}{1+z_{4}\left(t\right)}-300z_{5}\left(t\right)+\Gamma_{5}\left(t\right) \end{array} \end{array}\right.$$

**Figure 2:**
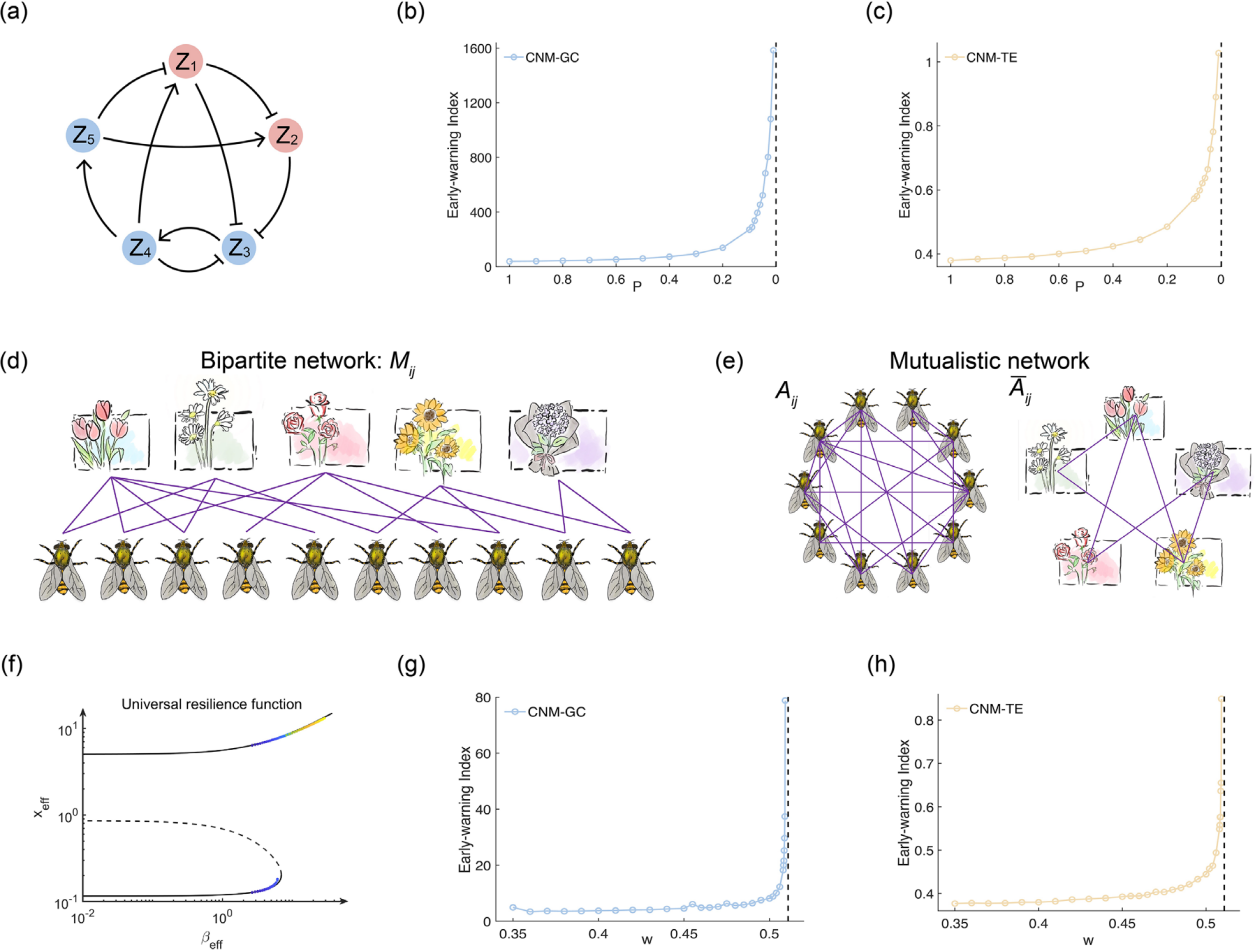
Early‐warning signals of the five‐gene genetic network and the mutualistic interaction network based on the causality marker. a) The network connection of the genetic network, with the theoretically verified DG (red nodes): {*Z*_1_, *Z*_2_}. d,e) The network connection of the mutualistic interaction network, where the separate networks (e) of the pollinators and the plants are generated from the bipartite interaction network (d). b,g) In the sense of GC, the marker increases rapidly when *P* approaches 0. c,h) In the sense of TE, the conclusion is similar. f) Universal resilience function obtained from our numerical simulation, which shows that Barabasi's dimension reduction of dynamics is reasonable.

In this genetic circuit, the eigenvalues of its corresponding discrete linearized system at the unique stable steady state $\bar{Z}=\left(1,0,1,3,2\right)$ are

(10) $$\begin{array}{c} \left(0.74^{\left|P\right|},0.55,0.37,0.20,0.14\right) \end{array}$$

which indicates that a phase transition occurs when *P* → 0. Additionally, we identify *z*_1_, *z*_2_ as the DG corresponding to the largest eigenvalue λ_max_ = 0.74^|*P*|^, which governs the phase transition (λ_max_ → 1 as |*P*| → 0) as described in ref. [6]. In theory, the causality strength will change thus can be detected by CNMs. To empirically investigate this property, we conduct numerical simulations. The results reveal that both marker values exhibit pronounced trends, offering early‐warning signals for the impending phase transition (see **Figure** **2**a–c).

#### 2.3.2 Ecological Mutualistic Interaction Network

In addition to bifurcations resulting from individual parameter changes, it is also essential to consider the occurrence of bifurcation when the interaction undergoes variations. A typical mutualistic interaction network comprises two distinct species such as pollinators, denoted as *PO*_*j*_ with *j* = 1, …, *n*, and plants, denoted as *PL*_*i*_ with *i* = 1, …, *m*. Their interactions are characterized by bipartite relationships represented by the matrix ${\left(M_{ij}\right)}_{\begin{array}{c} 1\leq i\leq n\\ 1\leq j\leq m \end{array}}$ (Figure 2d). After that, we can construct two distinct mutualistic networks by establishing connections between pairs of plants and pollinators derived from the *M*_*ij*_ matrix, following the methodology outlined in [21]:

(11) $$\begin{array}{cc} & A_{ij}=\frac{\sum_{k=1}^{m}M_{ik}M_{jk}}{\sum_{s=1}^{n}M_{sk}},\;1\leq i,j\leq n\\ & {\bar{A}}_{ij}=\frac{\sum_{k=1}^{n}M_{ki}M_{kj}}{\sum_{s=1}^{m}M_{km}},\;1\leq i,j\leq m \end{array}$$

Here, *A*_*ij*_ (1 ⩽ *i*, *j* ⩽ *n*) denotes the interaction strength within the pollinators network, while ${\bar{A}}_{ij}\;\left(1\leq i,j\leq m\right)$ denotes the interaction strength within the plants network (Figure 2e). Notably, the dynamical influence of one species on another is implicitly encapsulated within the parameters *A*_*ij*_ or ${\bar{A}}_{ij}$. Consequently, we can construct the dynamics of both the plants' network and the pollinators' network as follows (we only display the pollinators' network):^[^ ^21^, ^24^, ^25^ ^]^

(12) $$\begin{array}{cc} \frac{dx_{i}}{dt}= & s\left[B_{i}+x_{i}\left(1-\frac{x_{i}}{K_{i}}\right)\left(\frac{x_{i}}{C_{i}}-1\right)\right.\\ & +\left.\sum_{j=1}^{n}A_{ij}\frac{x_{i}x_{j}}{D_{i}+E_{i}x_{i}+H_{j}x_{j}}\right]+\Gamma_{i}\left(t\right),\\ & i=1,\cdots ,n \end{array}$$

In this dynamic, the first term accounts for logistic growth, encompassing factors such as the Allee effect and a constant influx attributed to migration. These factors are characterized by parameters *B*_*i*_, *K*_*i*_, *C*_*i*_, *D*_*i*_, *E*_*i*_, *H*_*i*_, and a scaling parameter *s* (where *s* = 1 in the work by Barabasi). The interaction term quantifies the symbiotic influence of each population *x*_*j*_ on *x*_*i*_, with its strength determined by the parameter *A*_*ij*_. This interaction term saturates as the population sizes become sufficiently large.

Detailed investigations of such networks have been conducted by Gao et al.^[^ ^21^, ^24^, ^25^ ^]^ Their research findings have revealed that when the parameters in Equation (12) exhibit node‐independence, with values such as *B*_*i*_ = *B*, *C*_*i*_ = *C*, *D*_*i*_ = *D*, *E*_*i*_ = *E*, *H*_*i*_ = *H*, and *K*_*i*_ = *K*, the high‐dimensional dynamics can be effectively reduced to one‐dimensional resilient dynamics through the application of mean‐field approximation:

(13) $$\begin{array}{c} \frac{dx_{\text{eff}}}{dt}=s\left[B+x_{\text{eff}}\left(1-\frac{x_{\text{eff}}}{K}\right)\left(\frac{x_{\text{eff}}}{C}-1\right)+\beta_{\text{eff}}\frac{x_{\text{eff}}^{2}}{D+\left(E+H\right)x_{\text{eff}}}\right] \end{array}$$

In this context, we denote $x_{\text{eff}}=\frac{1^{⊤}Ax}{1^{⊤}A1}$ as the efficient state, while β_eff_ represents a critical parameter referred to as the universal resilient state. Actually, β_eff_ serves to aggregate the influence of the interaction matrix A. Specifically, within the framework of mean‐field approximation, we can express β_eff_ as $\beta_{\text{eff}}=\frac{1^{⊤}A^{2}1}{1^{⊤}A1}$.^[^ ^21^ ^]^ Subsequently, we can solve the ODE Equation (13) that governs the dynamics of *x*_eff_ and β_eff_. This equation yields β_eff_ = β_eff_(*x*_eff_), which is regarded as a resilience function, describing the appropriate behavior of the stochastic evolution (Figure 2f). Barabasi and colleagues have observed a bifurcation phenomenon occurring when the interaction strengths are perturbed globally as *A*_*ij*_ → *w*_*ij*_ *A*_*ij*_. In this scenario, when both the efficient state *x*_eff_ and the universal resilient state β_eff_ simultaneously reach a threshold denoted as $x_{\text{eff}}^{c}$ and $\beta_{\text{eff}}^{c}$, respectively, defined as:

(14) $$\begin{array}{c} {\left.\frac{\partial f(\beta_{\text{eff}}^{c},x_{\text{eff}})}{\partial x_{\text{eff}}}\right|}_{x_{\text{eff}}^{c}}=0\;f\left(\beta_{\text{eff}}^{c},x_{\text{eff}}^{c}\right)=0 \end{array}$$

the system undergoes a transition from either a bistable or monostable state to the other. Specifically, the stability of one steady state, indicative of “low population density”, changes to either manifest or vanish.

In our numerical simulations, we have considered a specific scenario where the pollinators are considered invasive alien species, necessitating strict population control measures to maintain a small population size. Consequently, it becomes crucial to generate an early‐warning signal before the low population steady state loses its stability. To adapt our framework effectively to this context, we have intentionally disregarded the stochastic of *w*_*ij*_ and introduced a weak Gaussian white noise component into the dynamics to incorporate stochastic effects. In our initial parameter selection, as defined in Table S2 (Supporting Information), we aimed to establish stability in the high state while rendering the low state unstable. Subsequently, we deliberately set *w*_*ij*_ = *w* ≡ 0.3 to restore stability to both states. We systematically increased this global weight adjustment to examine the parameters that lead to instability in the low state. It is worth noting that under the influence of the noise term, the threshold is not a fixed constant but exhibits fluctuations within a small range around *w* = 0.52. This observation implies that as the low state becomes increasingly unstable, invasive alien species may proliferate, posing a significant threat to the ecological system.

Our analysis of the time series data obtained from simulations allows us to calculate the CNMs under both GC and TE measures, revealing a consistent surge in marker values preceding the bifurcation (see Figure 2d–h). Furthermore, we calculate the efficient state *x*_eff_ and its corresponding universal resilient state β_eff_ using simulations, as outlined in Equation (13) (Figure 2f). In instances where the system exhibits monostability for smaller values of *w* or bistability for larger values of *w*, our approximations of *x*_eff_ and β_eff_ consistently cluster near the resilience function. This observation underscores the rationality and effectiveness of Barabasi's dimension reduction approach to capturing the system's dynamics.^[^ ^21^ ^]^ In summary, our results demonstrate the versatility of the CNMs in detecting early‐warning signals for systems undergoing bifurcations, whether they are induced by single parameter changes, or global parameter changes, as exemplified by ecological mutualistic interaction networks. Notably, early‐warning signals in such multifactorial systems pose a unique challenge due to the unfixed nature of their tipping points, influenced by a multitude of parameters. These complex, multifactorial dynamics may not be effectively detected by conventional early‐warning signals. However, the CNMs provide a precise and comprehensive signal, as it encompasses a range of intricate and implicit influencing factors inherent in causality indices. This makes it well‐suited for detecting bifurcations driven by multifactorial influences.

#### 2.3.3 Turing Diffusion Interaction Network

Lastly, we explore the efficacy of the CNMs in detecting spatial bifurcation, highlighting its versatile applications. Beyond morphogenesis theories in developmental biology, reaction‐diffusion systems with spatial heterogeneity are relevant in various contexts. A reaction‐diffusion system shows diffusion‐driven instability, or Turing instability, when the homogeneous steady state remains stable without diffusion but becomes unstable to spatial perturbations with diffusion present.

In biology, instability often refers to the situation where a uniform steady state destabilizes under small perturbations, leading to non‐uniform behavior of ecological significance. Examples include environmental heterogeneity in animal dispersal,^[^ ^26^ ^]^ reaction‐diffusion in anisotropic growth domains,^[^ ^27^ ^]^ spatial invasion modelling,^[^ ^28^ ^]^ and differential diffusion in plant root initiation.^[^ ^29^ ^]^ Spatial heterogeneity influences local instability conditions, modulates pattern size and wavelength, and localizes spike patterns. Here, we focus on the Turing bifurcations, a complex phenomenon where the system transitions from a homogeneous stable state to a non‐uniform stable state under specific conditions.

The predator–prey model, which represents many realistic biological phenomena, also displays spatially non‐uniform behavior during the Turing instability. It is proposed (In this work, we call it Turing network for convenience) by Beddington and DeAngelis in a form of [30]:

(15) $$\left\{\begin{array}{c} \begin{array}{cc} \frac{\partial H(t,x,y)}{\partial t}= & r\left(1-\frac{H}{K}\right)H-\frac{\beta H}{B+H+\omega P}P\\ & +D_{1}\Delta_{x,y}H+\Gamma_{1}\left(t\right), \end{array}\\ \begin{array}{cc} \frac{\partial P(t,x,y)}{\partial t}= & \frac{\varepsilon \beta H}{B+H+\omega P}P-\eta P\\ & +D_{2}\Delta_{x,y}P+\Gamma_{2}\left(t\right) \end{array} \end{array}\right.$$

where the Turing bifurcation occurs with significantly different diffusion coefficients *D*_1_ and *D*_2_. The parameters are set as *r* = 0.5, ε = 1, β = 0.6, *B* = 0.4, η = 0.25, ω = 0.4, and *D*_2_ = 1 with $\Delta_{x,y}=\partial_{x}^{2}+\partial_{y}^{2}$. The remaining parameters *D*_1_ and *K* control the spatial pattern.

The spatial pattern arises from the instability of the time‐invariant steady state H*(*x*, *y*) and P*(*x*, *y*) that satisfy:

(16) $$\left\{\begin{array}{c} \begin{array}{c} r\left(1-\frac{H^{*}}{K}\right)H^{*}-\frac{\beta H^{*}}{B+H^{*}+\omega P^{*}}P^{*}+D_{1}\Delta_{x,y}H^{*}=0, \end{array}\\ \begin{array}{c} \frac{\varepsilon \beta H^{*}}{B+H^{*}+\omega P^{*}}P^{*}-\eta P^{*}+D_{2}\Delta_{x,y}P^{*}=0 \end{array} \end{array}\right.$$

when *D*_1_ and *D*_2_ differ significantly, the spatially homogeneous structure breaks, producing patterns with different expression levels at different spatial locations. In numerical simulations, we divide the spatial range (*x*, *y*) ∈ [0, *R*_*x*_] × [0, *R*_*y*_] into 11 × 11 lattices, and treating each lattice as a variable. We then apply our detection procedure to this system in the sense of CNM‐GC and CNM‐TE.

In the study of the Turing networks, minor differences in parameter selection leads to significant variations in the system's dynamical behavior, particularly the emergence of the Turing–Hopf bifurcations.^[^ ^31^, ^32^ ^]^ Relevant researches about this system have illustrated the bifurcation conditions in the parameter space, with regions representing the different types of bifurcation behaviors the system may experience.^[^ ^31^ ^]^ Specifically, the area below the Hopf bifurcation line indicates that the system remains stable over time without oscillations; however, the area above suggests the onset of periodic oscillatory behavior. Similarly, the area below the Turing bifurcation line implies spatial uniformity in the system, while the area above indicates the formation of spatial patterns. After the Turing bifurcation, with parameter changes, the system may exhibit a variety of pattern formations. Near the bifurcation point, the system generally displays a spot pattern structure, while far from the bifurcation point, it typically shows a stripe pattern.^[^ ^33^, ^34^, ^35^ ^]^

Through the analysis of genetic networks and ecological networks, we find that bifurcations in the temporal dimension can be effectively detected by our CNM‐GC and CNM‐TE. However, the core of this subsection is to demonstrate that these markers can also detect spatial bifurcation phenomena such as Turing bifurcations. To this end, we select specific parameter combinations *D*_1_ = 0.01, *D*_2_ = 1. As the parameter *D*_1_ increases, the system gradually enters Turing bifurcation without accompanying Hopf bifurcation.

In terms of experimental design, we conduct simulation experiments for each set of parameters (*D*_1_, *D*_2_) for up to 800 seconds to ensure that the system's evolution is in the vicinity of equilibrium. We employ a common data aggregation strategy: Integrating continuous simulation data with a 1‐second time window, thereby treating the node distribution at each second as a snapshot of the data within that second. The rationale for this method is based on the assumption that over a sufficiently short time scale, the system's dynamical behavior exhibits a certain consistency, allowing us to approximate the node distribution characteristics throughout the simulation process through short‐time data aggregation.

**Figure 3:**
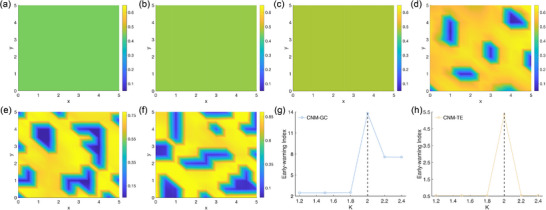
Early‐warning signals of the Turing diffusion interaction network based on the causality marker. a–f) The spatial broken phenomenon occurs near the Turing bifurcation. The transition parameter *K* is set as a) *K*=1.2, b) *K*=1.5, c) *K*=1.8, d, the pattern occurs) *K*=2, e) *K*=2.2, and f) *K*=2.4. g,h) In the sense of GC and TE, the CNMs increase rapidly only when the bifurcation occurs (*K* ≈ 2).

Furthermore, unlike the previous two systems where the DG was known, in this system with 121 lattices, we cannot a priori know the DG. Therefore, we use the *K*‐means clustering algorithm to distinguish between DG and NDG. **Figure** **3** shows the trend of changes in the CNM‐GC and CNM‐TE indices under different parameter settings. It can be observed from the figure that both markers reach their peaks near the middle, close to the parameters where Turing bifurcation occurs, which validates the effectiveness of the markers, that is, the system's tipping dynamics promote an increase in the CNM.

It is particularly noteworthy that the CNM‐GC shows a certain increase after the system crosses the steady‐state point compared to the parameter settings that have not crossed the steady‐state. This finding reveals that the system's regulatory mechanisms and temporal evolution characteristics may have undergone significant changes after bifurcation. It is not difficult to see that the construction of CNMs based on causal relationships is universal and applicable, not only effectively detecting temporal bifurcation phenomena but also revealing the formation of spatial patterns through spatial causal relationships, such as in the parameter area close to the occurrence of Turing bifurcation.

In summary, we tested the effectiveness of CNMs on genetic networks, ecological networks, and Turing networks, respectively. They represent the tipping points of internal dynamics of a single node, the interactions between nodes, and the spatial interactions (usually caused by different levels of diffusion), accordingly. In these validations, both CNM‐GC and CNM‐TE showed consistently accurate and consistent results, rendering by the fact that the module of exactly one eigenvalue of the discrete evolution matrix tending from 1^−^ to 1. However, in real datasets, their dynamics exhibit more complex characteristics, leading to various difficult to detect tipping points, such as using traditional DNB, and the identification of CNM‐GC and CNM‐TE may be more abundant.

### 2.4 Validations on Real‐World Datasets

**Figure 4:**
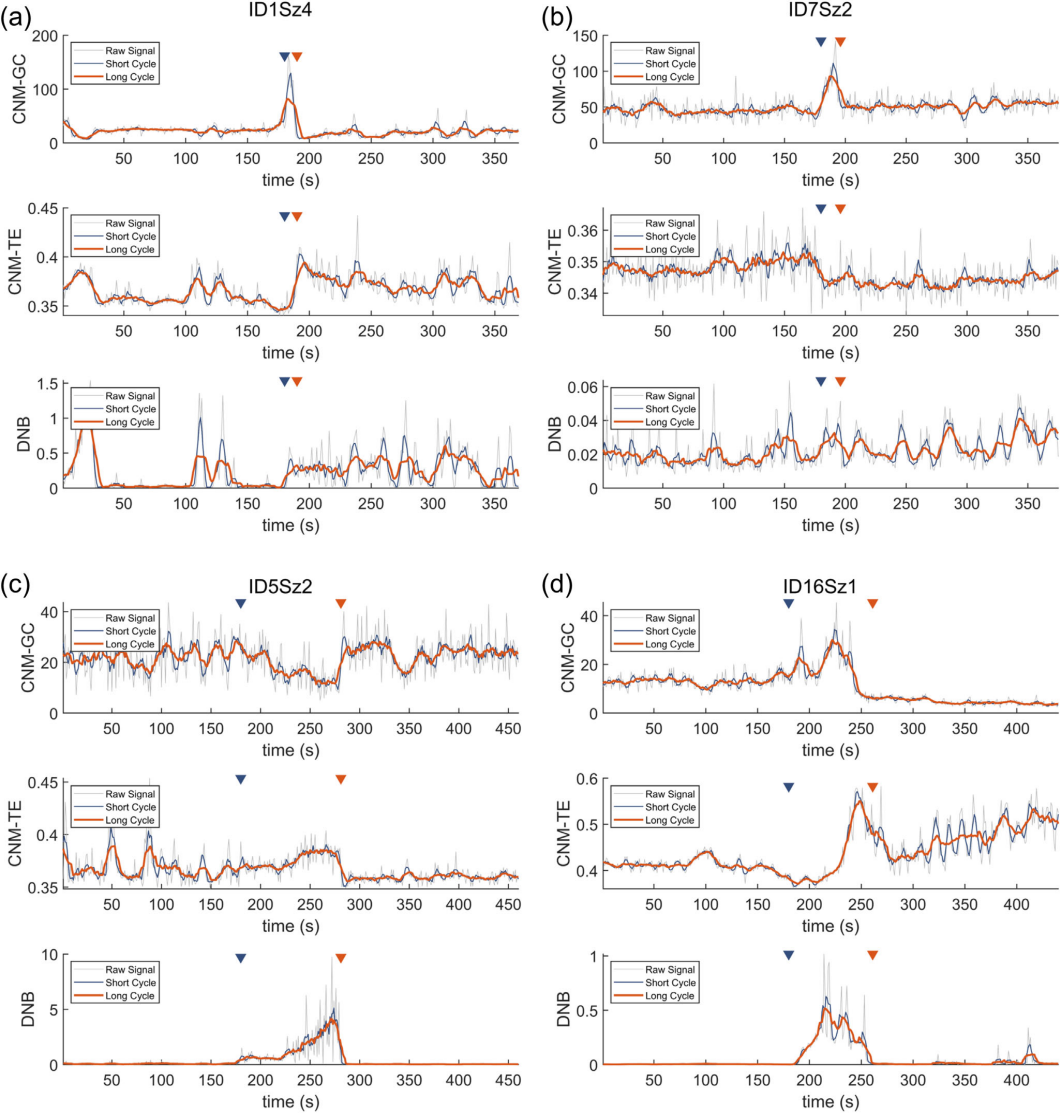
Tipping point detection results for the iEEG short‐term dataset. Patient IDs and seizure serial numbers in panels (a), (b), (c), and (d) correspond to ID1Sz4, ID7Sz2, ID5Sz2, and ID16Sz1, respectively. Each panel contains three subplots arranged vertically, displaying results from CNM‐GC, CNM‐TE, and DNB, respectively. Blue and red triangles in each subplot indicate seizure onset and termination, respectively. Each time series encompasses the period from 3 min before seizure onset to 3 min after seizure termination. The gray, blue, and red lines in each subplot represent the original marker/raw signal, 5 s moving average/short cycle, and 12 s moving average/long cycle, respectively.

Although real‐world datasets often have high complexity and their dynamics are difficult to construct, fortunately, mathematical theories such as the center manifold theorem ensure that the dynamics of the system near bifurcation points may be confined to a low‐dimensional manifold. Therefore, network markers similar to DNB can reveal potential dynamical bifurcation phenomena in real‐world datasets to a certain extent. However, the application of these methods to epilepsy data is still limited, mainly because the complex biological critical phenomena represented by epilepsy cannot be simply quantified using a few models. In this section, we apply the two types of CNMs we have constructed to human iEEG data from epilepsy patients,^[^ ^36^, ^37^ ^]^ in the hope of verifying the practicality and operability of the CNMs framework. Compared with the DNB, our metric is more sensitive in detecting the interaction relationship between nodes and can explain the dynamic bifurcation caused by causality in both linear and non‐linear senses. Therefore, this method provides a new perspective and evaluation criteria to understand the neurodynamic characteristics of complex diseases such as epilepsy, and provides theoretical support for personalized clinical diagnosis and treatment, as shown in **Figure** **4** and **Table** **1**.

**Table 1:** The Accuracy of different combinations of markers.

Markers	All Valid	CNMs	DNB	CNM‐GC only	CNM‐TE only
Accuracy [%]	43	92	79	68	74

#### 2.4.1 Datasets and Candidate Network Markers Introduction

Epilepsy is a chronic disorder that affects brain function, characterized by abnormal and excessive neuronal discharges that lead to recurrent epileptic seizures. Numerous benchmark models in epilepsy research have revealed a common mechanism: epileptic seizures typically signify a critical transition in brain state.^[^ ^38^, ^39^, ^40^ ^]^ This transition is thought to be associated with a tipping point, where the brain rapidly shifts from a normal state to a seizure state.^[^ ^41^, ^42^, ^43^ ^]^ In the natural world, critical transitions represent pivotal moments where the behavior of a system undergoes a fundamental change, and the theory of dynamical bifurcations provides a theoretical foundation and analytical toolkit for such phenomena. This theoretical framework aids in understanding and predicting changes in behavioral patterns as a system approaches a tipping point. When applied to epilepsy research, the theory of dynamical bifurcations enables the identification and analysis of key neurodynamic changes that may precipitate epileptic seizures, offering new perspectives and methods for the prediction and intervention of seizure events.

In this part, we choose the iEEG dataset to detect the epilepsy seizures (see the detailed introduction of the datasets in Supporting Information). Meanwhile, we select DNB, CNM‐GC, and CNM‐TE as candidates for seizure prediction and employ a unified assessment standard to measure the performance of these markers in warning of epilepsy. We apply the DG and NDG to DNB and CNMs, to assess their effectiveness on the dataset with a unified standard. The division of DG and NDG on real datasets is mainly determined by the variance of the nodes, stemming from the assurance that the variance of the DG tends to infinity near the critical point. Therefore, in the epilepsy dataset, we take the average of the variance of each node during the onset period and then use the *K*‐means clustering algorithm to provide group division. Note that no matter how the division is made, this division has subjectivity, but we further detect and obtain the consistent performance using different clustering algorithms^[^ ^44^, ^45^, ^46^, ^47^, ^48^ ^]^ (see Supporting Information). Of note, the calculation of the DNB, where

(17) $$\text{DNB(Net)}:=\frac{{\mathrm{SD}}_{d}\cdot {\mathrm{PCC}}_{d}}{{\mathrm{PCC}}_{o}}$$

is based on several key metrics, like the variance during the seizure, that quantify the dynamics within and between DG and NDG. Specifically, the DNB is calculated using the following parameters:

a) SD_*d*_: The average standard deviation of the nodes within the DG;
b) PCC_*d*_: The average absolute value of Pearson correlation coefficient among nodes within the DG;
c) PCC_*o*_: The average absolute value of Pearson correlation coefficient between nodes in the DG and those in the NDG.

#### 2.4.2 Result Analysis for iEEG Dataset

In this section, we investigate the performance differences of various markers on the same dataset. The results indicate that the CNMs show higher accuracy rather than DNB. Moreover, the combination of signals from the two distinct causal network markers, CNM‐GC and CNM‐TE, provides additional information about epileptic seizures, that is, we can preliminarily classify epileptic seizures in terms of causality using causality indicators.

First, DNB, as an efficient early‐warning method, completes this warning through the variance of nodes and the PCC between nodes. However, as an early‐warning tool, the effectiveness of DNB is not always reliable, especially when the nodes themselves are subject to significant noise interference. The variance term in the DNB fluctuates dramatically, greatly reducing its detection power. However, CNMs such as CNM‐GC and CNM‐TE are designed without considering the node's own role, thus they possess superior noise resistance. An example can be found in Supporting Information (Extra variance diagram of ID7Sz2, which means the 2‐nd seizure of the 7‐th patient in the dataset, in Supporting Information). Furthermore, the PCC indicator is not sensitive to minor changes in node interactions, leading to the DNB potentially being insufficient to detect early‐warning signals in some cases. The CNMs use a more sensitive causal relationship for critical state early‐warning, reflected by the higher accuracy of CNMs than DNB (Table 1). Specifically, the CNMs method has shown considerable effects in about 92% of the cases, which is higher than the overall effect of DNB in about 79%. In several seizures such as ID1Sz4 and ID7Sz2 in Figure 4a,b, the CNMs capture potential early‐warning information more effectively than DNB. At this time, the DNB shows an unstable pattern of oscillation, while the CNMs clearly distinguish the warning peaks. Therefore, due to their noise resistance, CNM‐GC and CNM‐TE still serve as powerful tools for predicting epileptic seizures when DNB fails.

Here, we first summarize the advantages, limitations, and prospects of CNMs and DNB in detecting epilepsy. Firstly, CNM‐GC and CNM‐TE have noise resistance. This is a significant advantage over traditional DNB methods, meaning that CNMs can maintain stable prediction performance even in data with higher system noise or unclear changes in PCC. Second, CNM‐GC and CNM‐TE bring a comprehensive evaluation. We can infer the causal patterns of critical dynamics of epileptic seizures in both linear and non‐linear senses through two different causality indicators. Furthermore, CNMs is versatile. The calculation framework of this marker can be directly extended to other causal senses, such as EE, which can help further comprehensively identify seizure patterns. As an attempt to identify epilepsy from a new perspective and evaluation criteria, the traditional early‐warning identification methods of CNM‐GC and CNM‐TE may fail in some special cases due to the complexity of complex disease neurodynamics. This may be due to causal explosion in other senses besides GC and TE and may be explained by the CSD model of causality indicators. In addition, there are still many aspects to be improved in the multi‐index epilepsy classifier, such as indicator selection and further experiments. At the same time, CNMs may have broad prospects for development in the clinical treatment of epilepsy. If these indicators are integrated into existing epilepsy prediction models, on the one hand, they provide causal network markers of epilepsy for researchers, and on the other hand, they may be combined with multiple indicators to provide more personalized management and treatment strategies for epilepsy patients. In general, CNM‐GC and CNM‐TE demonstrate robust performance on short‐term epileptic datasets exhibiting critical dynamics, and their effectiveness can be further validated on real‐world critical transitions, such as earthquakes^[^ ^49^ ^]^ (see further applicability of CNMs in Supporting Information).

#### 2.4.3 Neurological Analysis on the iEEG Dataset

We also discover that the tipping dynamics of epilepsy may have different causal patterns. In terms of dynamical causality (DC), GC relies on linear regression and is thus considered representative of linear causality, whereas TE, which incorporates nonlinear information transfer, is viewed as capturing nonlinear causality.^[^ ^11^ ^]^ The rise of CNM‐GC and CNM‐TE in Figure 4a indicates that the epileptic seizure in this case is a mixture of linear and non‐linear causality. There are cases of epilepsy caused solely by one type of causality. As shown in Figure 4b, in this case, CNM‐GC identifies effective early‐warning, while CNM‐TE fails, which indicates that the epileptic seizures in this case may be more affected by direct and linear neural activity patterns; As shown in Figure 4c, in this case, CNM‐TE identifies effective early‐warning, while CNM‐GC fails. The seizures in this case may originate from more complex and non‐linear neural activity patterns. This difference indirectly confirms the complexity of the mechanisms that trigger epileptic seizures and inspires us to conduct a more in‐depth discussion and analysis of the critical dynamics of epilepsy. This may be because some high‐dimensional critical dynamics properties of epilepsy cannot be characterized by low‐dimensional critical dynamics, which is also worth further exploration in future work. However, in 43% cases (Table), the CNM‐GC, CNM‐TE, and DNB indicators all show a clear upward trend, consistently demonstrating a certain sense of early‐warning in the critical dynamics of epilepsy, such as Figure 4d.

For epilepsy datasets, different seizure samples may exhibit distinct patterns, and the CNMs framework provides a perspective for epilepsy pattern classification within the DC framework. An important question thus arises: what is the association between dynamical causality and neural dynamics in these epileptic patterns? We designed a dynamics estimation experiment to investigate the potential association between dynamics and neural patterns, with analysis of potential epileptic mechanisms provided (See Supporting Information). However, theoretical development remains incomplete regarding causal patterns ahead of critical transitions, requiring further investigation in future studies. In addition, our causal classification align with some previous experimental validations, such as the critical slowing down (CSD) in iEEG.^[^ ^50^ ^]^ In some rare individual cases, it reveals the existence of CSD phenomena (see Supporting Information for introduction of CSD and our identification example such as ID5Sz1). This indicates that the mode of epilepsy seizure shows more than two causal patterns we explored. It is required that we need to make a comprehensive judgment based on various indicators, to provide more accurate early‐warning signals for epileptic seizures in the future.

## 3 Conclusion

In this study, we introduce a framework of causal network markers, called CNMs, for identifying general bifurcation phenomena in the dynamical evolution of complex systems. In the framework, we construct a functional form of CNMs that reflects the strength of system causality. We introduce two markers, CNM‐GC and CNM‐TE, representing the “linear causality” and the “non‐linear causality”, respectively.

To experimentally verify the efficacy of CNM‐GC and CNM‐TE, we use the data produced by computational benchmark models and collected from the real‐world systems. Precisely, we consider the data from three benchmark models: the genetic network, the ecological network, and the Turing diffusion interaction network. They respectively represent the internal, interaction, and diffusion effects reaching criticality, encompassing three types of typical critical phenomena in the common stage. The results indicate that CNMs have significant predictive effects on both temporal and spatial bifurcation models. Meanwhile, the real‐world dataset we use comes from a epilepsy dataset, which is typically considered to have complex dynamical properties. We found that in this situation, the identification of CNMs composed of causality indicators with different meanings may be inconsistent, and the combination of two causal network markers has high predictive accuracy, which provides a new causal perspective for early‐warning and identification of epilepsy.

As a causal‐oriented unidirectional markers, CNMs framework focuses on the interactions between nodes and is not affected by the internal dynamics of node variance. It can capture more information than the PCC between nodes, as in DNB, and are resistant to noise and highly sensitive. The CNMs also detect different kinds of temporal‐spatial tipping points, making it simple and versatile. At the same time, it is scalable and flexible, where many of the causality indicator can be introduced into this framework, including the spatial causality^[^ ^51^ ^]^ and the machine learning technique.^[^ ^52^ ^]^ We have listed the practical guidance on introducing suitable causal markers into CNMs framework in the Supporting Information. When multiple causality indicators are selected, CNMs generally provide a comprehensive evaluation, which is beneficial to identifying more complex dynamics of the system so that it is applicable to early‐warning detection with broad application prospects. Recently, some improved approaches based on DNB, introducing spatiotemporal information or relative entropy, have been proposed to construct the early‐warning signal of critical transition in various situation like earthquake or infectious disease epidemics.^[^ ^49^, ^53^, ^54^, ^55^ ^]^ We believe that, these methods can motivate the CNMs framework, from an information theory perspective, to construct more comprehensive analysis and quantitative characterization for early warning of critical transitions.

## 4 Experimental Section

### Statistical Analysis

Numerical results were shown as the mean with standard error. All the results were performed by MATLAB.

## Conflict of Interest

The authors declare no conflict of interest.

## Supporting information

Supporting Information

## Data Availability

The data that support the findings of this study are available from the corresponding author upon reasonable request.
