# Supplementary material for: Utilizing Causal Network Markers to Identify Tipping Points ahead of Critical Transition
Source: Adv Sci (Weinh). 2025 Sep 5;12(42):e15732. doi: 10.1002/advs.202415732 (PMC12622457; doi:10.1002/advs.202415732)
Supplement: Supplementary file 1 — Supporting Information [file ADVS-12-e15732-s001.pdf]

SUPPORTING INFORMATION

Utilizing Causal Network Markers to Identify Tipping Points ahead of Critical Transition

Shirui Bian[1,2], Zezhou Wang[3], Siyang Leng[2,4], Wei Lin[1,2,3,5,6] and Jifan Shi[†2]

[1]School of Mathematical Sciences, Fudan University, Shanghai, 200433, China.
[2]Research Institute of Intelligent Complex Systems, Fudan University, Shanghai, 200433, China.
[3]Shanghai Center for Mathematical Sciences, Fudan University, Shanghai, 200433, China.
[4]Institute of AI and Robotics, College of Intelligent Robotics and Advanced Manufacturing, Fudan University, Fudan University, Shanghai, 200433, China.
[5]Shanghai Artificial Intelligence Laboratory, Shanghai, 200232, China.
[6]State Key Laboratory of Medical Neurobiology and MOE Frontiers Center for Brain Science, Fudan University, Shanghai, 200032, China.

[†] Correspondence: jfshi@fudan.edu.cn

Contents

1 Vanishment of Granger causality in the vicinity of tipping points 3

2 Pseudo-code for causal network markers (CNMs) 8

3 Effectiveness of CNMs under different clustering algorithms 10

4 Practical guidance on introducing suitable causality indicators into CNMs framework 11

5 Robustness of CNMs framework against noise perturbation 13

6 Technical details of the ecological network 14

7 Figures of CNMs on real-world earthquake dataset 15

8 Description of iEEG datasets 16

9 Association between dynamical causality and neural dynamics from epileptic patterns 18

10 Figures of CNMs and DNB's validation on iEEG 21

Code availability 33

Reference 34

1 Vanishment of Granger causality in the vicinity of tipping points

In the realm of multidimensional discrete dynamical systems, the evolution of the system with parameter $\boldsymbol{P}$ is governed by the equation

$$\boldsymbol{Z}^{t+1} = \boldsymbol{f}(\boldsymbol{Z}^t;\ \boldsymbol{P}), \tag{1}$$

where $\boldsymbol{Z}^t = (z_1^t, z_2^t, \dots, z_n^t)$ represents a vector of n components at time t, and $\boldsymbol{f}$ is a continuously differentiable vector function that possesses a non-trivial fixed point $\bar{\boldsymbol{Z}}$, satisfied the condition $\boldsymbol{f}(\bar{\boldsymbol{Z}}) = \bar{\boldsymbol{Z}}$. It is posited that there exists a critical parameter value $\boldsymbol{P}_c$ which serves as a tipping point, inducing a codimension-one bifurcation in the system as $\boldsymbol{P}$ approaches $\boldsymbol{P}_c$. The linearized form of the system in the vicinity of $\bar{\boldsymbol{Z}}$ is expressed as

$$\boldsymbol{X}^{t+1} = \boldsymbol{A}\boldsymbol{X}^t + \boldsymbol{\Gamma}^t, \tag{2}$$

where $\boldsymbol{X}^t$ is the deviation vector from the fixed point, defined as $\boldsymbol{X}^t = \boldsymbol{Z}^t - \bar{\boldsymbol{Z}}$, and $\boldsymbol{A}$ is the Jacobian matrix of $\boldsymbol{f}$ evaluated at $\bar{\boldsymbol{Z}}$, given by

$$\boldsymbol{A} = \left.\frac{\partial \boldsymbol{f}(\boldsymbol{Z};\ \boldsymbol{P})}{\partial \boldsymbol{Z}}\right|_{\boldsymbol{Z}=\bar{\boldsymbol{Z}}}. \tag{3}$$

The vector $\boldsymbol{\Gamma}^t$ encapsulates the higher-order terms after the linear approximation. To elucidate our findings, we consider a typical scenario where $\boldsymbol{A}$ is diagonalizable on $\mathbb{R}^{n\times n}$, and $\boldsymbol{A}$ can be decomposed as $\boldsymbol{A} = \boldsymbol{S}\boldsymbol{\Lambda}\boldsymbol{S}^{-1}$ with $\boldsymbol{S}, \boldsymbol{\Lambda} \in \mathbb{R}^{n\times n}$. After applying the transformation $\boldsymbol{Y}^t = \boldsymbol{S}^{-1}\boldsymbol{X}^t$, we obtain that:

$$\begin{aligned} \boldsymbol{S}\boldsymbol{Y}^{t+1} &= \boldsymbol{A}\boldsymbol{S}\boldsymbol{Y}^t + \boldsymbol{\Gamma}^t = (\boldsymbol{S}\boldsymbol{\Lambda}\boldsymbol{S}^{-1})\boldsymbol{S}\boldsymbol{Y}^t + \boldsymbol{\Gamma}^t, \\ \boldsymbol{Y}^{t+1} &= \boldsymbol{\Lambda}\boldsymbol{Y}^t + \boldsymbol{S}^{-1}\boldsymbol{\Gamma}^t. \end{aligned} \tag{4}$$

Here, $\boldsymbol{Y}^t = (y_1^t, y_2^t, \dots, y_n^t)$ satisfies a diagonal dynamics. In the subsequent discussion, we postulate y_1 to be the only element in the "dominant group". Specifically, as $\boldsymbol{P} \to \boldsymbol{P}_c$, the variance of y_1 tends to infinity, while the Pearson correlation coefficient between y_1 and the other variables approaches 0. With this assumption, we now proceed to calculate the Granger causality between any two variables x_i and x_j in the original phase space.

In the context of the analysis concerning the causal relationship from $x_i^t = s_{i1}y_1^t + \cdots + s_{in}y_n^t$ to $x_j^t = s_{j1}y_1^t + \cdots + s_{jn}y_n^t$, we delineate two distinct models: the H_0-model and the H_1-model, which are articulated as

$$\begin{cases} H_0: \ x_i^{t+1} = a x_i^t + \xi_1, \\ H_1: \ x_i^{t+1} = b x_i^t + c x_j^t + \xi_2, \end{cases} \tag{5}$$

where $a,\ b,\ c$ are the coefficients of the linear fitting, and $\xi_1,\ \xi_2$ are the corresponding residue. Within the H_0-model, the expectation of ξ_1^2 is derived as follows:

$$\begin{aligned}\mathbb{E}[\xi_1^2] =& \min_a \mathbb{E}\left[(x_i^{t+1} - a x_i^t)^2\right] \\ =& \min_a \left(\mathbb{E}[(x_i^{t+1})^2] - 2a\mathbb{E}[x_i^{t+1}x_i^t] + a^2\mathbb{E}[(x_i^t)^2]\right) \\ =& \mathbb{E}[(x_i^{t+1})^2] - \frac{\mathbb{E}[x_i^{t+1}x_i^t]^2}{\mathbb{E}[(x_i^t)^2]}.\end{aligned} \tag{6}$$

This expression represents the minimum achievable variance of the prediction error under the H_0-model, achieved by optimizing the parameter a.

Transitioning to the H_1-model, we employ the method of least squares to calculate $\mathbb{E}[\xi_2^2]$:

$$\begin{aligned}\mathbb{E}[\xi_2^2] =& \min_{b,c} \mathbb{E}\left[(x_i^{t+1} - b x_i^t - c x_j^t)^2\right] \\ =& \min_{b,c} \mathbb{E}\left[(x_i^{t+1})^2 - 2\begin{pmatrix} x_i^t x_i^{t+1} & x_j^t x_i^{t+1}\end{pmatrix}\begin{pmatrix} b \\ c\end{pmatrix} + \begin{pmatrix} b & c\end{pmatrix}\begin{pmatrix}(x_i^t)^2 & x_i^t x_j^t \\ x_j^t x_i^t & (x_j^t)^2\end{pmatrix}\begin{pmatrix} b \\ c\end{pmatrix}\right] \\ =& \min_{b,c}\left[\mathbb{E}[(x_i^{t+1})^2] - 2\begin{pmatrix}\mathbb{E}[x_i^t x_i^{t+1}] \\ \mathbb{E}[x_j^t x_i^{t+1}]\end{pmatrix}^\top \begin{pmatrix} b \\ c\end{pmatrix} + \begin{pmatrix} b \\ c\end{pmatrix}^\top \begin{pmatrix}\mathbb{E}[(x_i^t)^2] & \mathbb{E}[x_i^t x_j^t] \\ \mathbb{E}[x_j^t x_i^t] & \mathbb{E}[(x_j^t)^2]\end{pmatrix}\begin{pmatrix} b \\ c\end{pmatrix}\right].\end{aligned} \tag{7}$$

When it reaches the minimum, the derivative with respect to $(b, c)^\top$ must vanish, which yields

$$-2\begin{pmatrix}\mathbb{E}[x_i^t x_i^{t+1}] \\ \mathbb{E}[x_j^t x_i^{t+1}]\end{pmatrix} + 2\begin{pmatrix}\mathbb{E}[(x_i^t)^2] & \mathbb{E}[x_i^t x_j^t] \\ \mathbb{E}[x_j^t x_i^t] & \mathbb{E}[(x_j^t)^2]\end{pmatrix}\begin{pmatrix} b^* \\ c^*\end{pmatrix} = \begin{pmatrix} 0 \\ 0\end{pmatrix} \Longrightarrow \begin{pmatrix} b^* \\ c^*\end{pmatrix} = \boldsymbol{A}^{-1}\boldsymbol{v}, \tag{8}$$

where $\boldsymbol{A}$ and $\boldsymbol{v}$ are defined as:

$$\boldsymbol{A} = \begin{pmatrix}\mathbb{E}[(x_i^t)^2] & \mathbb{E}[x_i^t x_j^t] \\ \mathbb{E}[x_j^t x_i^t] & \mathbb{E}[(x_j^t)^2]\end{pmatrix},\ \boldsymbol{v} = \begin{pmatrix}\mathbb{E}[x_i^t x_i^{t+1}] \\ \mathbb{E}[x_j^t x_i^{t+1}]\end{pmatrix}. \tag{9}$$

Substitute Eq. (8) and (9) into Eq. (7), we obtain (notice that $\boldsymbol{A} = \boldsymbol{A}^\top$):

$$\begin{aligned}\mathbb{E}[\xi_2^2] =& \mathbb{E}[(x_i^{t+1})^2] - 2(\boldsymbol{A}^{-1}\boldsymbol{v})^\top\boldsymbol{v} + (\boldsymbol{A}^{-1}\boldsymbol{v})^\top\boldsymbol{A}(\boldsymbol{A}^{-1}\boldsymbol{v}) \\ =& \mathbb{E}[(x_i^{t+1})^2] - 2\boldsymbol{v}^\top\boldsymbol{A}^{-1}\boldsymbol{v} + \boldsymbol{v}^\top\boldsymbol{A}^{-1}\boldsymbol{A}(\boldsymbol{A}^{-1}\boldsymbol{v}) \\ =& \mathbb{E}[(x_i^{t+1})^2] - 2\boldsymbol{v}^\top\boldsymbol{A}^{-1}\boldsymbol{v} + \boldsymbol{v}^\top\boldsymbol{A}^{-1}\boldsymbol{v} \\ =& \mathbb{E}[(x_i^{t+1})^2] - \boldsymbol{v}^\top\boldsymbol{A}^{-1}\boldsymbol{v} \\ =& \mathbb{E}[(x_i^{t+1})^2] - \begin{pmatrix}\mathbb{E}[x_i^t x_i^{t+1}] \\ \mathbb{E}[x_j^t x_i^{t+1}]\end{pmatrix}^\top \begin{pmatrix}\mathbb{E}[(x_i^t)^2] & \mathbb{E}[x_i^t x_j^t] \\ \mathbb{E}[x_j^t x_i^t] & \mathbb{E}[(x_j^t)^2]\end{pmatrix}^{-1}\begin{pmatrix}\mathbb{E}[x_i^t x_i^{t+1}] \\ \mathbb{E}[x_j^t x_i^{t+1}]\end{pmatrix}.\end{aligned} \tag{10}$$

In summary, we obtain:

$$\begin{cases}\mathbb{E}[\xi_1^2] = \mathbb{E}[(x_i^{t+1})^2] - \dfrac{\mathbb{E}[x_i^{t+1}x_i^t]^2}{\mathbb{E}[(x_i^t)^2]}, \\ \mathbb{E}[\xi_2^2] = \mathbb{E}[(x_i^{t+1})^2] - \begin{pmatrix}\mathbb{E}[x_i^t x_i^{t+1}] \\ \mathbb{E}[x_j^t x_i^{t+1}]\end{pmatrix}^\top \begin{pmatrix}\mathbb{E}[(x_i^t)^2] & \mathbb{E}[x_i^t x_j^t] \\ \mathbb{E}[x_j^t x_i^t] & \mathbb{E}[(x_j^t)^2]\end{pmatrix}^{-1} \begin{pmatrix}\mathbb{E}[x_i^t x_i^{t+1}] \\ \mathbb{E}[x_j^t x_i^{t+1}]\end{pmatrix},\end{cases} \tag{11}$$

where we introduce the notations $\mathbb{E}[y_i^t y_j^t] = \rho_{ij}$ and $\boldsymbol{S}^{-1}\boldsymbol{\Gamma}^t = \boldsymbol{\varepsilon}^t$. Given the premise that y_1^t is the only dominant variable, with $\rho_{11} \to +\infty$ and and $PCC(y_1^t, y_i^t) \to 0$ as $\boldsymbol{P} \to \boldsymbol{P}_c$, it is justifiable to focus on terms in Eq. (11) that include ρ_{11}. We proceed under the assumption that

$$\begin{aligned}&\mathbb{E}[(x_i^t)^2] = k_1\rho_{11} + B_1,\ \mathbb{E}[(x_j^t)^2] = k_2\rho_{11} + B_2,\ \mathbb{E}[x_i^t x_j^t] = k_3\rho_{11} + B_3, \\ &\mathbb{E}[(x_i^{t+1})^2] = k_4\rho_{11} + B_4,\ \mathbb{E}[x_i^t x_i^{t+1}] = k_5\rho_{11} + B_5,\ \mathbb{E}[x_j^t x_i^{t+1}] = k_6\rho_{11} + B_6.\end{aligned} \tag{12}$$

These assumptions allow for the calculation of the coefficients k_i through the relationships $x_i^t = \sum_k s_{ik} y_k^t$ and $x_i^{t+1} = \sum_k s_{ik} y_k^{t+1} = \sum_k s_{ik}(\lambda_k y_k^t + \varepsilon_k)$. Assuming that B_i do not significantly change as the system approaches the tipping point, they can be treated as constants:

$$\begin{cases}k_1 = s_{i1}^2,\ k_2 = s_{j1}^2,\ k_3 = s_{i1}s_{j1}, \\ k_4 = \lambda_1^2 s_{i1}^2,\ k_5 = \lambda_1 s_{i1}^2,\ k_6 = \lambda_1 s_{i1} s_{j1}.\end{cases} \tag{13}$$

In H_0-model, we obtain

$$\begin{aligned}\mathbb{E}[\xi_1^2] =& \frac{\mathbb{E}[(x_i^{t+1})^2]\mathbb{E}[(x_i^t)^2] - \mathbb{E}[x_i^{t+1}x_i^t]^2}{\mathbb{E}[(x_i^t)^2]} \\ =& \frac{(k_4\rho_{11} + B_4)(k_1\rho_{11} + B_1) - (k_5\rho_{11} + B_5)^2}{k_1\rho_{11} + B_1} \\ =& \frac{(\lambda_1^2 s_{i1}^2 \cdot s_{i1}^2 - (\lambda_1 s_{i1}^2)^2)\rho_{11}^2 + (k_4B_1 + k_1B_4 - 2k_5B_5)\rho_{11} + B_1B_4 - B_5^2}{k_1\rho_{11} + B_1} \\ =& \frac{(k_4B_1 + k_1B_4 - 2k_5B_5)\rho_{11} + B_1B_4 - B_5^2}{k_1\rho_{11} + B_1},\end{aligned} \tag{14}$$

while in H_1-model we obtain

$$
\begin{aligned}
\mathbb{E}[\xi_2^2] =& \mathbb{E}[(x_i^{t+1})^2] - \frac{1}{\mathbb{E}[(x_i^t)^2]\mathbb{E}[(x_j^t)^2] - \mathbb{E}[x_i^t x_j^t]^2} \Big[\mathbb{E}[(x_j^t)^2]\mathbb{E}[x_i^t x_i^{t+1}]^2 \\
& + \mathbb{E}[(x_i^t)^2]\mathbb{E}[x_j^t x_i^{t+1}]^2 - 2\mathbb{E}[x_i^t x_j^t]\mathbb{E}[x_i^t x_i^{t+1}]\mathbb{E}[x_j^t x_i^{t+1}]\Big] \\
=& \frac{1}{\mathbb{E}[(x_i^t)^2]\mathbb{E}[(x_j^t)^2] - \mathbb{E}[x_i^t x_j^t]^2} \Big[\mathbb{E}[(x_i^{t+1})^2]\Big(\mathbb{E}[(x_i^t)^2]\mathbb{E}[(x_j^t)^2] - \mathbb{E}[x_i^t x_j^t]^2\Big) \\
& - \Big(\mathbb{E}[(x_j^t)^2]\mathbb{E}[x_i^t x_i^{t+1}]^2 + \mathbb{E}[(x_i^t)^2]\mathbb{E}[x_j^t x_i^{t+1}]^2 - 2\mathbb{E}[x_i^t x_j^t]\mathbb{E}[x_i^t x_i^{t+1}]\mathbb{E}[x_j^t x_i^{t+1}]\Big)\Big] \\
=& \frac{1}{(k_1\rho_{11} + B_1)(k_2\rho_{11} + B_2) - (k_3\rho_{11} + B_3)^2} \Big[(k_4\rho_{11} + B_4)\Big((k_1\rho_{11} + B_1) \\
& \cdot(k_2\rho_{11} + B_2) - (k_3\rho_{11} + B_3)^2\Big) - (k_2\rho_{11} + B_2)(k_5\rho_{11} + B_5)^2 - (k_1\rho_{11} + B_1) \\
& \cdot(k_6\rho_{11} + B_6)^2 + 2(k_3\rho_{11} + B_3)(k_5\rho_{11} + B_5)(k_6\rho_{11} + B_6)\Big].
\end{aligned} \tag{15}
$$

In the denominator, the coefficient of ρ_{11}^2 is $k_1k_2 - k_3^2 = s_{i1}^2 s_{j1}^2 - (s_{i1}s_{j1})^2 = 0$, and the coefficient of ρ_{11} is $k_1B_2 + k_2B_1 - 2k_3B_3$. While in the numerator, the coefficient of ρ_{11}^3 is $k_4(k_1k_2 - k_3^2) - k_2k_5^2 - k_1k_6^2 + 2k_3k_5k_6 = 0$, the coefficient of ρ_{11}^2 is

$$
\begin{aligned}
& k_4(k_1B_2 + k_2B_1 - 2k_3B_3) - (2k_2k_5B_5 + B_2k_5^2) \\
& - (2k_1k_6B_6 + B_1k_6^2) + 2(k_3k_5B_6 + k_5k_6B_3 + k_6k_3B_5) \\
=& B_1(k_2k_4 - k_6^2) + B_2(k_1k_4 - k_5^2) + B_3(2k_5k_6 - 2k_3k_4) \\
& + B_5(2k_6k_3 - 2k_2k_5) + B_6(2k_3k_5 - 2k_1k_6) \\
=& 0.
\end{aligned} \tag{16}
$$

Finally, the coefficient of ρ_{11} is

$$
\begin{aligned}
& k_4(B_1B_2 - B_3)^2 + B_4(k_1B_2 + k_2B_1 - 2k_3B_3) - (k_2B_5^2 + 2k_5B_2B_5) \\
& - (k_1B_6^2 + 2k_6B_1B_6) + 2(k_3B_5B_6 + k_5B_6B_3 + k_6B_3B_5).
\end{aligned} \tag{17}
$$

In summary, we simplify $\mathbb{E}[\xi_2^2]$ as

$$
\begin{aligned}
\mathbb{E}[\xi_2^2] =& \frac{1}{(k_1B_2 + k_2B_1 - 2k_3B_3)\rho_{11} + C_1} \\
& \cdot\Big[\Big(k_4(B_1B_2 - B_3)^2 + B_4(k_1B_2 + k_2B_1 - 2k_3B_3) - (k_2B_5^2 + 2k_5B_2B_5) \\
& - (k_1B_6^2 + 2k_6B_1B_6) + 2(k_3B_5B_6 + k_5B_6B_3 + k_6B_3B_5)\Big)\rho_{11} + C_2\Big]
\end{aligned} \tag{18}
$$

where C_1 and C_2 are constants. We now aim to demonstrate that when $s_{i1} = 0$ and $s_{j1} \neq 0$, implying that the j-th variable is related to the dominant variable y_1^t while the i-th is not, $\mathbb{E}[\xi_2^2] \to \mathbb{E}[\xi_1^2]$. Consequently, the Granger causality strength from x_j^t

to x_i^t, quantified by $\mathrm{GC}_{x_j \to x_i} = \log \frac{\mathbb{E}[\xi_1^2]}{\mathbb{E}[\xi_2^2]}$, approaches 0. This outcome stems from the fact that when $s_{i1} = 0$ and $s_{j1} \neq 0$, we have $k_1 = k_3 = k_4 = k_5 = k_6 = 0$, thus:

$$\begin{cases} \mathbb{E}[\xi_1^2] = \dfrac{B_1 B_4 - B_5^2}{B_1}, \\ \mathbb{E}[\xi_2^2] = \dfrac{k_2(B_4 B_1 - B_5^2)\rho_{11} + C_2}{k_2 B_1 \rho_{11} + C_1} \\ \qquad \to \dfrac{B_1 B_4 - B_5^2}{B_1} = \mathbb{E}[\xi_1^2] \quad (\rho_{11} \to +\infty). \end{cases} \tag{19}$$

For analogous reasons, we discuss the other three scenarios:

(a) $s_{i1} \neq 0$ and $s_{j1} = 0$, then $k_2 = k_3 = k_6 = 0$. Simplification of $\mathbb{E}[\xi_1^2]$ and $\mathbb{E}[\xi_2^2]$ yields:

$$\begin{aligned} \mathbb{E}[\xi_1^2] =& \frac{(k_4 B_1 + k_1 B_4 - 2k_5 B_5)\rho_{11} + B_1 B_4 - B_5^2}{k_1 \rho_{11} + B_1} \\ \longrightarrow& \frac{k_4 B_1 + k_1 B_4 - 2k_5 B_5}{k_1} \\ & (\rho_{11} \to +\infty), \end{aligned} \tag{20}$$

$$\begin{aligned} \mathbb{E}[\xi_2^2] =& \frac{1}{k_1 B_2 \rho_{11} + C_1} \Big[\Big(k_4 (B_1 B_2 - B_3)^2 + k_1 B_4 B_2 \\ & - 2k_5 B_2 B_5 - k_1 B_6^2 + 2k_5 B_6 B_3 \Big) \rho_{11} + C_2 \Big] \\ \longrightarrow& \frac{k_4 (B_1 B_2 - B_3)^2 + k_1 B_4 B_2 - 2k_5 B_2 B_5 - k_1 B_6^2 + 2k_5 B_6 B_3}{k_1 B_2} \\ & (\rho_{11} \to +\infty). \end{aligned} \tag{21}$$

Thus $\mathrm{GC}_{x_j \to x_i}$ tends to a constant.

(b) $s_{i1} \neq 0$ and $s_{j1} \neq 0$, for the same reason, $\mathrm{GC}_{x_j \to x_i}$ tends to a constant.

(c) $s_{i1} = 0$ and $s_{j1} = 0$, then $k_1 = k_2 = \cdots = k_6 = 0$ which means all the coefficient of ρ_{11} is 0. As $\rho_{11} \to +\infty$, the values of $\mathbb{E}[\xi_1^2]$ and $\mathbb{E}[\xi_2^2]$ are not influenced. As the result, $\mathrm{GC}_{x_j \to x_i}$ is invariant.

In conclusion, we have demonstrated that as the system reaches a tipping point, the causality from variables in the "dominant group" (DG) to those in the "non-dominant group" (NDG) tends to 0, while the causality from variables in NDG to those in DG tends to a constant. Simultaneously, the causality between variables in NDG remains invariant.

2 Pseudo-code for causal network markers (CNMs)

Algorithm 1 Partition of DG and NDG

Input: Node i, $i = 1, ..., N$; Node connections set $\mathcal{E}$; Variance temporal series $\sigma_i(1 : T)$ on node i, $i = 1, ..., N$; Partition begin time t_1; Partition end time t_2.
Output: DG set $\mathcal{G}_1$; NDG set $\mathcal{G}_2$; Directed connections pair $\mathcal{E}_{\mathcal{G}_1 \to \mathcal{G}_2}$ from DG to NDG.

for $i = 1 : N$ **do**
  $\overline{\sigma}_i \leftarrow \text{mean}(\sigma_i(t_1 : t_2))$ ▷ Calculate the mean of variance of each node
end for
$idx(1 : N)$, C_1, $C_2 \leftarrow \text{kmeans}([\overline{\sigma}_1, ..., \overline{\sigma}_N], 2)$ ▷ $idx(i) \in \{1, 2\}$ represents the cluster index of node i, C_1 and C_2 represent two centroids, and we let $C_1 \geq C_2$
$\mathcal{G}_1$, $\mathcal{G}_2 \leftarrow \emptyset$
for $i = 1 : N$ **do**
  if $idx(i) = 1$ **then**
    Add i to $\mathcal{G}_1$
  else if $idx(i) = 2$ **then**
    Add i to $\mathcal{G}_2$
  end if
end for
$\mathcal{E}_{\mathcal{G}_1 \to \mathcal{G}_2} \leftarrow \emptyset$
for $j = 1 : \text{size}(\mathcal{G}_1)$ **do**
  for $k = 1 : \text{size}(\mathcal{G}_2)$ **do**
    if $(\mathcal{G}_1(j), \mathcal{G}_2(k)) \in \mathcal{E}$ **then**
      Add $(\mathcal{G}_1(j), \mathcal{G}_2(k))$ to $\mathcal{E}_{\mathcal{G}_1 \to \mathcal{G}_2}$
    end if
  end for
end for
return $\mathcal{G}_1$, $\mathcal{G}_2$, and $\mathcal{E}_{\mathcal{G}_1 \to \mathcal{G}_2}$

Note: K-means clustering is a classic algorithm, and it is widely applied. In Algorithm 1, $[idx,\ C] = \text{kmeans}(X, k)$ performs K-means clustering to divide the observations of the $N \times 1$ data matrix X into k clusters. It returns an $N \times 1$ vector idx containing the cluster index for each observation, and an $k \times 1$ vector C represents the position of the Centroids of each cluster.

Algorithm 2 CNMs framework

Input: Vector of distribution data $\boldsymbol{p}_i(t)$ of node i at discrete time t, $t = 1, ..., T$; DG set $\mathcal{G}_1$; NDG set $\mathcal{G}_2$; Directed connections pair $\mathcal{E}_{\mathcal{G}_1 \to \mathcal{G}_2}$ from DG to NDG.
Output: CNM-GC $M_{GC}(1:T-1)$ and CNM-TE $M_{TE}(1:T-1)$

```
M_GC(1 : T − 1) = zeros(1 : T − 1)
M_TE(1 : T − 1) = zeros(1 : T − 1)
for j = 1 : size(E_{G1→G2}) do
    (x, y) = E_{G1→G2}(j)                ▷ x is a node in DG, and y is a node in NDG
    GC_{x→y}(1 : T − 1) = zeros(1 : T − 1)
    TE_{x→y}(1 : T − 1) = zeros(1 : T − 1)
    for t = 1 : T − 1 do
        GC_{x→y}(t) = Granger_causality (p_y(t), p_y(t + 1), p_x(t))
        TE_{x→y}(t) = Transfer_entropy (p_y(t), p_y(t + 1), p_x(t))
    end for
    M_GC(1 : T − 1) = M_GC(1 : T − 1) + GC_{x→y}(1 : T − 1)
    M_TE(1 : T − 1) = M_TE(1 : T − 1) + TE_{x→y}(1 : T − 1)
end for
M_GC(1 : T − 1) = size(E_{G1→G2}) / M_GC(1 : T − 1)
M_TE(1 : T − 1) = size(E_{G1→G2}) / M_TE(1 : T − 1)
return M_GC(1 : T − 1) and M_TE(1 : T − 1)
```

Note: Granger_causality $\left(\boldsymbol{p}_y(t),\, \boldsymbol{p}_y(t+1),\, \boldsymbol{p}_x(t)\right)$ and Transfer_entropy $\left(\boldsymbol{p}_y(t),\, \boldsymbol{p}_y(t+1),\, \boldsymbol{p}_x(t)\right)$ are two traditional causalities. Please refer to the Github link at the end of this supporting information for the reference code. Generally speaking, the early warnings can be implemented by setting suitable thresholds for CNM-GC and CNM-TE.

3 Effectiveness of CNMs under different clustering algorithms

In the CNMs framework, we use the K-means algorithm to divide nodes into DG and NDG based on the average variance of nodes during the given period. Here, we would like to compare and discuss alternative clustering algorithms to validate the capability of the algorithm to identify DG and NDG.

Specifically, we conducted a comprehensive comparative study of five representative clustering approaches K-means, hierarchical clustering, density-based spatial clustering of applications with noise (DBSCAN), spectral clustering, and gaussian mixture model (GMM) applied to our iEEG seizure datasets. Notably, all algorithms achieved effective DG/NDG partitioning across all four clinical cases in Figure 4 of the main text, and are displayed in Figure S1.

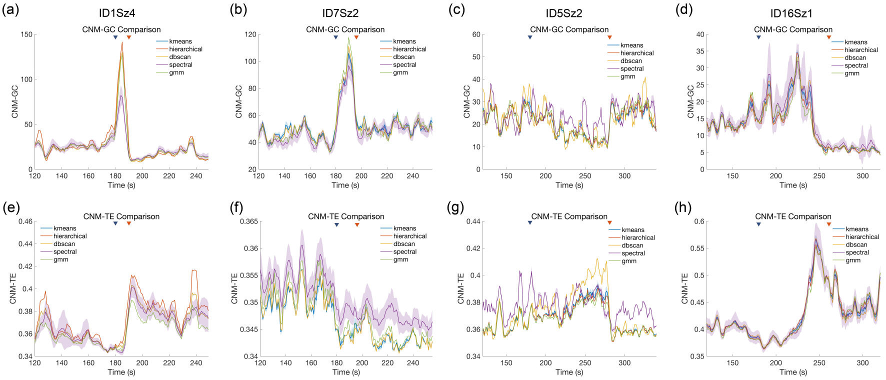

Figure S1. Comparative analysis of clustering algorithms of CNMs. (a-d) CNM-GC results and (e-h) CNM-TE results corresponding to four scenarios in Figure 4 of the main text. Solid lines represent 30-trial ensemble means for K-means, spectral clustering, and GMM, with shaded regions indicating ± 1 standard deviation. Deterministic results from hierarchical clustering and DBSCAN are also superimposed.

Although the principles of various algorithms are different, the variance of DG nodes in epilepsy data is significantly higher than that of NDG, resulting in similar classification results under different clustering algorithms. This phenomenon indicates that the dominance of variance differences limits the impact of clustering method selection on the final CNMs. In order to eliminate the randomness of the clustering algorithm itself, we provided CNMs confidence diagram of K-means, spectral clustering, and GMM algorithms for 30 trials Figure S1, further verifying the robustness of the clustering method (There is no stochasticity of hierarchical clustering and DBSCAN for the given condition). These systematic comparisons demonstrate that our framework's effectiveness stems from the variance contrast during critical transitions, rather than specific clustering.

4 Practical guidance on introducing suitable causality indicators into CNMs framework

The causality indicators for CNMs are interpreted within the dynamical causality (DC) framework, with detailed definitions available in reference [1]. The selection of appropriate causality indicators requires careful evaluation of both their applicability across different scenarios and their adherence to the causal vanishing property at tipping points.

For applicable scenarios, the choice of causality indicators in the CNMs framework is guided by three key considerations: system dynamics characteristics, data availability constraints, and computational tractability. GC proves most effective for linear systems with separable interactions, offering computational efficiency while assuming Gaussian noise distributions. For nonlinear systems without non-separability constraints, TE provides broader applicability despite its requirement for more extensive datasets. When dealing with non-separable systems, convergent cross mapping/embedded causality (CCM/EC) achieves an optimal balance between accuracy and scalability through local linear approximations in delay embedding spaces. In cases of highly nonlinear and non-separable systems, embedding entropy (EE) robustly captures complex dependencies via mutual information analysis, albeit with substantially increased computational demands. Table S1 presents a comprehensive synthesis of these markers within the DC framework, highlighting their problem-specific strengths. This unified taxonomy enables systematic selection:prioritize GC for simplicity in linear contexts, TE for moderate nonlinearity with sufficient data, CCM/EC for scalable non-separable systems, and EE for high-precision analysis of intricate nonlinear transitions. Hybrid approaches combining complementary markers (e.g., CNM-GC and CNM-TE in our article) may further enhance adaptability across diverse scenarios.

Table S1. Applicable scenarios of typical DC [1].

	GC	TE	CCM/EC	EE
Modeling Space	Original Space (X_i^t, X_j^t)	Original Space (X_i^t, X_j^t)	Delay Embedding Space $(X_i^t, X_j^{t,*})$	Delay Embedding Space $(X_i^t, X_j^{t,*})$
Mapping	Linear	Nonlinear	Local Linear	Nonlinear
Solvability of non-separability problem	No	No	Yes	Yes
H_0 model index	$\ln \mathrm{Var}(\xi_0)$	$H(x_i^t \mid x_i^{t-1}, \ldots, x_i^{t-p})$	$\lvert \mathrm{PCC}(\hat{X}_j^t, X_j^{t,*} \mid H_1) \rvert$	$\lvert \mathrm{MI}(X_j^{t,*}, X_i^{t,NN} \mid H_1) \rvert$
H_1 model index	$\ln \mathrm{Var}(\xi_1)$	$H(x_i^t \mid x_i^{t-1}, \ldots, x_i^{t-p}, x_j^{t-1}, \ldots, x_j^{t-p})$	$\lvert \mathrm{PCC}(\hat{X}_j^t, X_j^{t,*} \mid H_0) \rvert = 0$	$\lvert \mathrm{MI}(X_j^{t,*}, X_i^{t,NN} \mid H_0) \rvert = 0$

Regarding verification of the causal vanishing property, while we have not directly validated this for all markers (including CCM/EC and EE), such verification

typically follows straightforward procedures. As a toy model, we implemented a two-dimensional stochastic system to confirm this property for CNM-EE, described by the system:

$$\begin{cases} \dot{x} = px + y + \Gamma_x \\ \dot{y} = -10y + \Gamma_y \end{cases}.$$

By systematically varying the parameter p from -1 to 0^- (through values -0.8, -0.6, -0.4, -0.2, -0.1, -0.05, -0.01), we induced a system bifurcation where x belongs to the DG and y to the NDG. From the Takens' embedding theorem, a high-dimensional observation of partial variables can reconstruct the original dynamics, building the theoretical foundation of EE. The embedding theorem guarantees that a high-dimensional observation of variable x can reconstruct the original dynamics. Here, we set a time-delay observation of x as $h(x) = (x(t), x(t-\tau), x(t-2\tau), x(t-3\tau), x(t-4\tau))$, where $\tau = 1$, and calculated its mutual information in this embedding space (i.e., the EE of the original variable). As the result shows in Figure S2 we detect a significant increase of CNM-EE, which directly demonstrate the "causal vanishing" property.

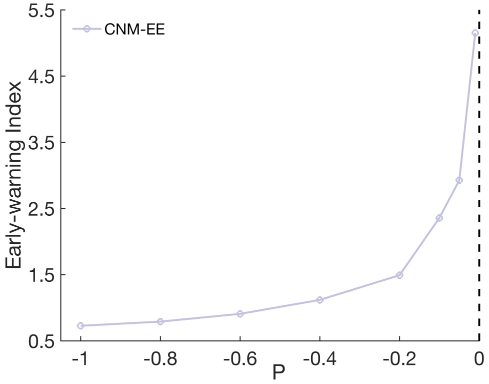

Figure S2. The performance of CNM-EE in a 2-dimensional toy model. When the system undergoes the tipping point, the "causality vanishing" also holds for EE, thus CNM-EE is also applicable for a significant increase.

5 Robustness of CNMs framework against noise perturbation

Here, we design an investigation to test the ability of CNM-GC to sustain robustness against noise perturbations in the five-genetic network. The results show that, corresponding to the obvious variance of DNB, the CNM-GC changes little under different noise level. Specifically, our investigation is designed as below: We set various noise strength $D = 10^{-4}$, 10^{-5}, 10^{-6}, 10^{-7} in the five-genetic network and perform the CNM-GC and DNB. The robustness under different noise level is characterized as the scale variance of these two markers. The results in Figure S3 indicate that, the DNB changes rapidly, while the CNM-GC holds the same scale under various D. This is because the calculation of causality, such as the linear fitting in GC, holds for scaling stability rather than the calculation of variance and correlation coefficient in the DNB theoretically. Consequently, the CNMs framework is benefit for setting a fixed threshold in a specific system for its scaling stability, as we claimed in the manuscript.

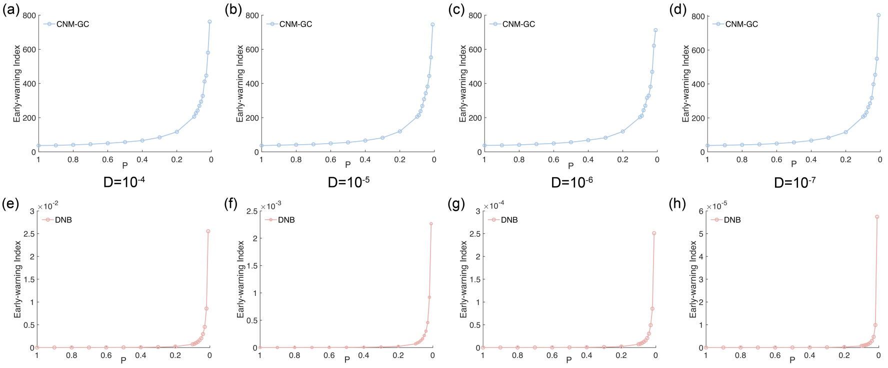

Figure S3. The indices performance under various noise strengths. We set the noise strength as $D = 10^{-4}, 10^{-5}, 10^{-6}, 10^{-7}$ for CNM-GC (a, b, c, d) and DNB (e, f, g, h) in the five-gene genetic network. The scale of CNM-GC is almost invariant, while that of DNB changes significantly, indicating higher noise resistance of CNM-GC.

6 Technical details of the ecological network

Table S2. Parameter chosen in ecological mutualistic interaction network.

Parameters	Values	Parameters	Values
B	0.3	C	2
D	2	E	0.9
H	0.1	K	2.5
m	10	n	5
s	80		
Mutual interaction matrix M $\begin{pmatrix} 10 & 10 & 10 & 0 & 10 & 0 & 0 & 10 & 0 & 0 \\ 1 & 0 & 1 & 0 & 0 & 1 & 0 & 0 & 0 & 0 \\ 0 & 1 & 0 & 1 & 0 & 0 & 1 & 0 & 1 & 0 \\ 0 & 0 & 0 & 0 & 0 & 1 & 0 & 1 & 0 & 1 \\ 0 & 0 & 0 & 0 & 0 & 0 & 0 & 0 & 1 & 1 \end{pmatrix}$			

It is worth mentioning that the reason why we choose $s = 80$ is that when the system is about to enter a critical state, only one eigenvalue in the system can tend to ± 1, to ensure that our indicator can effectively send an early warning signal. At the same time, there should be significant differences between other eigenvalues and the principal eigenvalue, otherwise, the impact of other eigenvalues on the index is probably to be huge. This flaw occurs not only in the global causality model we have established but also in DNB. DNB not only requires that the principal eigenvalue is significantly different from other eigenvalues, but also requires that the system noise is sufficiently small. The same index constructed based on the causality only considers the unidirectional relationships between nodes, so the conditional restriction of small noise is removed in a sense. In the future, it may be a meaningful extension direction to consider the high-dimensional bifurcation, that is, the existence of multiple principal eigenvalues.

7 Figures of CNMs on real-world earthquake dataset

To demonstrate the generalizability of the CNMs framework, we have applied it to earthquake early warning using Global Navigation Satellite System (GNSS)-based geodetic monitoring. As a cost-effective geodetic technique, GNSS leverages satellites' high-precision positioning to detect minute crustal displacement variations across multiple locations simultaneously [2, 3]. As shown in Figure S4, we tested CNMs' performance on typical 2018 earthquakes in central and southern Alaska, with data sources and preprocessing consistent with literature [3].

The results demonstrate that CNMs remain applicable for early warning of both individual earthquakes and seismic clusters, achieving robust performance across typical events. Similar to their performance in epileptic data, CNMs primarily captured "linear" and "nonlinear" causal patterns in seismic data a reflection of Earth's highly nonlinear, non-stationary, and complex dynamics [3]. Notably, we also observed complex patterns like critical slowing down (CSD), suggesting potential shared causal mechanisms between these systems that warrant further investigation in the future work.

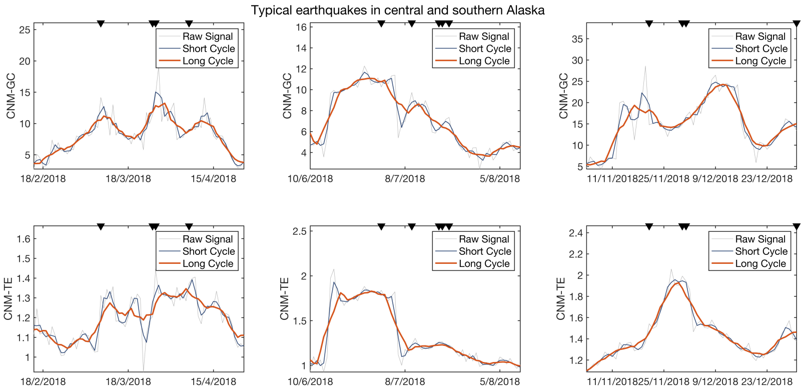

Figure S4. The performance of CNMs in typical earthquakes in central and southern Alaska, USA in 2018. The black triangle markers represent earthquake of magnitude≥5, while the short cycle and long cycle represent the 3-day and 7-day moving averages of the corresponding makers, respectively.

8 Description of iEEG datasets

The accurate detection and analysis of epileptic seizures are crucial for effective diagnosis and treatment. Intracranial electroencephalography (iEEG) technology provides a high-resolution view of brain electrical activity, offering valuable insights into the complex dynamics of epileptic seizures. iEEG, also known as electrocorticography or stereotactic EEG, can be performed through subdural strip or grid electrocorticography (ECoG) implanted in the subdural space, or via stereotaxic EEG (sEEG) that penetrates the brain to precisely target deeper regions such as the hippocampus, without the need for open-skull surgery [4].

In this research domain, the Sleep-Wake-Epilepsy Center (SWEC) at the Inselspital Bern, in collaboration with the Integrated Systems Laboratory of the ETH Zurich, has made a significant contribution by providing a carefully selected short-term iEEG dataset [5, 6]. These datasets, designed for research and educational purposes, serve as invaluable resources for the development and evaluation of seizure detection algorithms.

The short-term iEEG dataset compiles 100 anonymized records from 16 patients with drug-resistant epilepsy, offering a rich and diverse collection for in-depth analysis. Captured through strip, grid, and depth electrodes, these recordings undergo rigorous digital processing, including band-pass filtering and artifact rejection, to ensure high-quality data. We then subsequently test our causal marker on this short-term dataset.

The short-term iEEG datasets are accessible via http://ieeg-swez.ethz.ch/, in which signals were recorded in the brain through strip electrode, grid electrode and depth electrode. After 16-bit analog-to-digital conversion, the fourth-order Butterworth filter is applied to filter the data between 0.5 and 150 Hz, and the forward and backward filtering methods are used to reduce the phase distortion [5, 6]. The data is written to a disk at the rate of 512 Hz. All iEEG records manually excluded channels that were continuously damaged by artifacts. Each record comprises a 3-minute preictal period (before the onset of an epileptic seizure), the ictal period (during the onset of an epileptic seizure), and a 3-minute postictal period (after the onset of an epileptic seizure).

For our analysis, we established a 1-second time window, segmenting each short-term dataset into second-long intervals. This approach is based on the assumption that dynamical patterns exhibited by the system over sufficiently brief time frames remain analogous, making the 1-second window negligible compared to the entire dataset spanning hundreds of seconds. This methodology is reminiscent of our treatment of Turing patterns, where data within the time window are treated in parallel to derive an approximate probability distribution. The distinction lies in the fact that while Turing patterns detect spatial bifurcations, our analysis is concerned with temporal bifurcations.

In addition, in some rare cases of the iEEG dataset, such as ID5Sz1, our results may indicate the existence of CSD in the CNMs of system dynamics [7]. CSD refers to the possibility that variables may be pushed to another equilibrium state due to noise interference before reaching a stable or unstable equilibrium point when the system dynamically evolves to a bifurcation point. Perhaps specific markers lose

their peak under the interference of certain factors, such as false causality or indirect causality, but their essence can be explained by CSD. The manifestation of causal CSD effects in the human brain network is still a question worth studying about. In addition, in some cases of the iEEG dataset, such as ID5Sz1, our CNMs do not exhibit a peak shape, but slowly change from one state to another, perhaps due to CSD [7] in the dynamics of the epileptic system.

In natural systems, sudden state changes, including seizures, can theoretically be caused by critical transitions. CSD refers to the situation where the time to return to the equilibrium state becomes longer when the system's dynamic evolution approaches the bifurcation point. If the system is gradually approaching the tipping point and the attraction basin around the equilibrium point can be approximated by linear stability analysis, CSD may occur. The effect of CSD is manifested in the increase in variance and autocorrelation of signals, which has been observed in many systems, including cell population collapse in bacterial culture and financial market crashes. Studying CSD in epilepsy datasets is crucial to supporting current theoretical models of seizure generation and general theories of brain dynamics, which can help predict seizures and adjust epilepsy treatment plans [7].

According to the speed of the system variable approaching the tipping point, CSD occurs on different time scales, and sometimes the critical transition time is sufficiently long. ID5Sz1 may correspond to a slow trend towards the tipping point in the system, and we cannot simply characterize the shifts in system state and the rate of system change from the variation of variance. However, CNMs are different in that they can distinguish some CSDs on large time scales.

9 Association between dynamical causality and neural dynamics from epileptic patterns

For epilepsy datasets, distinct seizure samples may exhibit diverse patterns, and the CNMs framework offers a systematic approach for classifying these patterns within the DC framework. However, the theoretical linkage between these causal patterns and the underlying neural dynamics or physiological mechanisms in epilepsy remains incomplete. To preliminarily bridge this gap, we conducted an exploratory analysis by segmenting iEEG data and estimating neural dynamics using linear approximations, providing tentative insights into their potential relationships.

Mathematically, experimental dataset are partitioned into 4-second $4f \times M$ segments, where $f = 512$ represents the sampling frequency and M indicates the number of electrode channels. For each i-th segment spanning time indices $t = 4(i-1)f+1, \dots, 4if$, we model the system's linear dynamics as:

$$\dot{\boldsymbol{x}}(t) = \boldsymbol{A}_i \boldsymbol{x}(t) + \boldsymbol{B}_i, \tag{22}$$

where $\boldsymbol{A}_i \in \mathbb{R}^{M\times M}$ represents the linear interaction matrix and $\boldsymbol{B}_i \in \mathbb{R}^{M\times 1}$ is the bias vector capturing low-order nonlinear effects. Both require estimation through the following least-squares optimization:

$$\min_{\boldsymbol{A}_i,\boldsymbol{B}_i} \sum_j \left\| \hat{\dot{\boldsymbol{x}}}\left(t_j^i\right) - \boldsymbol{A}_i \boldsymbol{x}\left(t_j^i\right) - \boldsymbol{B}_i \right\|_2^2, \tag{23}$$

where t_j^i corresponds to the j-th timestamp in the i-th segment. The derivative estimate $\hat{\dot{\boldsymbol{x}}}(t_j)$ is computed via central differencing:

$$\hat{\dot{\boldsymbol{x}}}\left(t_j^i\right) = \frac{\boldsymbol{x}\left(t_{j+1}\right) - \boldsymbol{x}\left(t_{j-1}\right)}{t_{j+1} - t_{j-1}}, \tag{24}$$

with boundary points ($j = 1$ and $j = 4f$) handled using forward/backward difference schemes, respectively.

To reformulate the problem, we define the augmented matrix $\boldsymbol{W} = \begin{bmatrix}\boldsymbol{A}_i & \boldsymbol{B}_i\end{bmatrix} \in \mathbb{R}^{M\times(M+1)}$, and:

$$\boldsymbol{D} = \left[\hat{\dot{\boldsymbol{x}}}\left(t_1^i\right) \ \cdots \ \hat{\dot{\boldsymbol{x}}}\left(t_{4f}^i\right)\right] \in \mathbb{R}^{M\times 4f},$$

$$\boldsymbol{Y} = \begin{bmatrix} \boldsymbol{x}\left(t_1^i\right) & \cdots & \boldsymbol{x}\left(t_{4f}^i\right) \\ 1 & \cdots & 1 \end{bmatrix} \in \mathbb{R}^{(M+1)\times 4f}.$$

The optimization problem in (23) can be re-expressed as:

$$\min_{\boldsymbol{W}} \|\boldsymbol{D} - \boldsymbol{W}\boldsymbol{Y}\|_F^2, \tag{25}$$

where $\|\cdot\|_F$ represents the Frobenius norm. Let $\langle \cdot, \cdot \rangle$ denote the matrices inner product. Taking the derivative with respect to $\boldsymbol{W}$:

$$\begin{aligned}\frac{\mathrm{d}}{\mathrm{d}\boldsymbol{W}}\|\boldsymbol{D}-\boldsymbol{W}\boldsymbol{Y}\|_F^2 &= \frac{\mathrm{d}}{\mathrm{d}\boldsymbol{W}}\langle \boldsymbol{D}-\boldsymbol{W}\boldsymbol{Y}, \boldsymbol{D}-\boldsymbol{W}\boldsymbol{Y}\rangle \\ &= \frac{\mathrm{d}}{\mathrm{d}\boldsymbol{W}}\left(\langle \boldsymbol{W}\boldsymbol{Y}, \boldsymbol{W}\boldsymbol{Y}\rangle - 2\langle \boldsymbol{D}, \boldsymbol{W}\boldsymbol{Y}\rangle + \langle \boldsymbol{D}, \boldsymbol{D}\rangle\right) \\ &= 2\boldsymbol{W}\boldsymbol{Y}\boldsymbol{Y}^\top - 2\boldsymbol{D}\boldsymbol{Y}^\top.\end{aligned}$$

Setting the derivative to zero yields the closed-form solution:

$$\boldsymbol{W} = \boldsymbol{D}\boldsymbol{Y}^\top \left(\boldsymbol{Y}\boldsymbol{Y}^\top\right)^{-1}.$$

After obtaining the optimal estimate above, we applied it to Figure 4 in the main text. It is worth noting that, the CNM-GC and CNM-TE patterns can be further categorized into four distinct scenarios during seizure onset: (a) CNM-GC and CNM-TE signals nearly synchronous emergence, (b) only CNM-GC dominates while CNM-TE fails, (c) only CNM-TE dominates while CNM-GC fails, and (d) CNM-GC and CNM-TE signals alternating dominance. To investigate the associations between these causal patterns and estimated dynamics, we analyzed the changes of relative sizes of $\|\boldsymbol{A}_i\|_F$ reflecting linear interactions, and $\|\boldsymbol{B}_i\|_F$ reflecting nonlinear bias, across these four scenarios, which as shown in Figure S5.

The results in Figure S5 indicate that, firstly, CNM-GC correlated with both $\|\boldsymbol{A}_i\|_F$ and $\|\boldsymbol{B}_i\|_F$. In scenario (a) and (b), peaks in $\|\boldsymbol{A}_i\|_F$ and $\|\boldsymbol{B}_i\|_F$ synchronize with CNM-GC signals. In scenario (c), the CNM-GC peak near $t = 200$ corresponds to a $\|\boldsymbol{A}_i\|_F$ peak and a minor $\|\boldsymbol{B}_i\|_F$ peak near $t = 200$. In addition, CNM-GC exhibits heightened sensitivity to $\|\boldsymbol{A}_i\|_F$ in early transitions. For instance, also in scenario (d), while $\|\boldsymbol{A}_i\|_F$ responds to the early CNM-GC peak near $t = 200$, it shows the trivial response to later CNM-GC peaks between $t = 200 \sim 250$. In scenario (c), despite CNM-GC signals exhibiting noisy fluctuations at the early stage, $\|\boldsymbol{A}_i\|_F$ and $\|\boldsymbol{B}_i\|_F$ still increase. Secondly, CNM-TE predominantly correlated with $\|\boldsymbol{B}_i\|_F$. For example, in scenario (a), the effect of CNM-TE overlaps with CNM-GC. In scenario (c), $\|\boldsymbol{B}_i\|_F$ aligns with CNM-TE peaks between $t = 250 \sim 300$. In scenario (d), CNM-TE primarily drives the elevation of $\|\boldsymbol{B}_i\|_F$ in the latter part of the entire peak under asynchronous signals.

From a physical interpretation perspective, the enhancement of CNM-GC reflects an increase in linearity within system interactions, thereby indirectly driving the elevation of the nonlinear bias term $\|\boldsymbol{B}_i\|_F$. Conversely, the enhancement of CNM-TE signifies the amplification of nonlinear dynamics, indicating a rise in low-order nonlinear terms $\|\boldsymbol{B}_i\|_F$. Intriguingly, the early-stage sensitivity of $\|\boldsymbol{A}_i\|_F$ to CNM-GC signals appears to foreshadow several features of neural dynamics during epileptic transitions. Specifically, in the early phase of seizures, the system exhibits weak nonlinearity with reinforced dynamical coupling, the structural changes of the system can be captured by $\boldsymbol{A}_i$, manifesting as an increase in $\|\boldsymbol{A}_i\|_F$. Statistically, however, the linearity of neural dynamics does not fully align with the "linearity" defined under the

DC framework. This mismatch may explain why CNM-GC signals appear as noise during early seizure onset in scenario (c) or as minor peaks in scenario (d). As the seizure progresses beyond this initial phase, the system transitions into strong nonlinearity with degraded dynamical coupling. Although linear correlations may persist statistically, resulting in residual CNM-GC signals, the physical meaning of $\boldsymbol{A}_i$ fails in this period. Consequently, $\|\boldsymbol{A}_i\|_F$ ceases to correlate with CNM-GC variations, reflecting a decoupling between linear statistical associations and true dynamical mechanisms.

Through the above discussion, we have tentatively studied the associations and differences between causal patterns and neural dynamics in the context of linear dynamics estimation. However, the true neural dynamics of epilepsy are far more complex, and establishing rigorous correspondences to neurophysiological mechanisms remains challenging. We believe this will be a very meaningful and worthwhile topic to explore further.

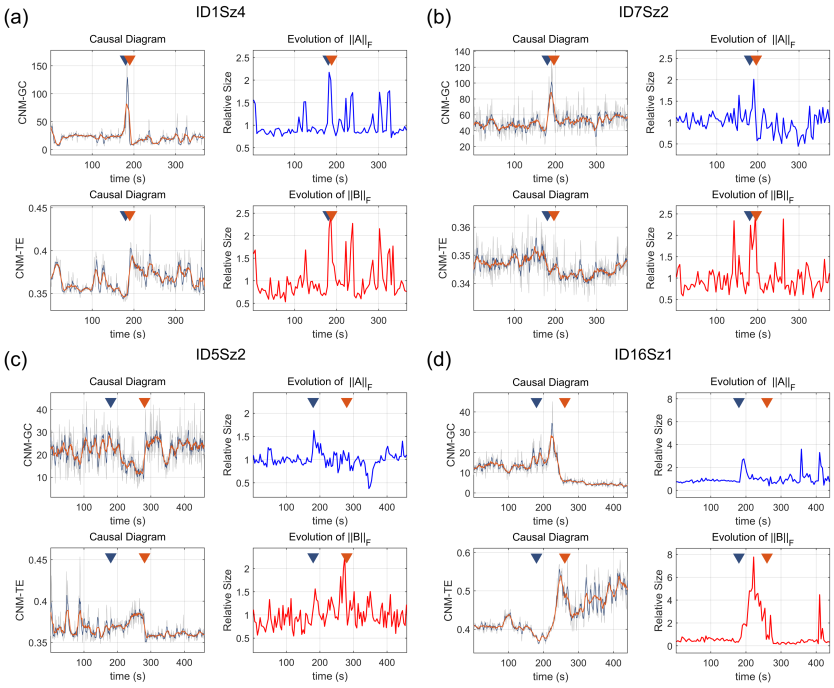

Figure S5. Comparison between CNMs results and linear approximations of neural dynamics. (a)-(d) correspond to the four epilepsy scenarios presented in Figure 4 of the main text, with left subplots showing causal diagrams and right subplots displaying linear dynamic estimations through temporal evolution of $\|\boldsymbol{A}\|_F$ (top) and $\|\boldsymbol{B}\|_F$ (bottom). The relative size represents their normalized quantities, i.e., original size divided by their means.

10 Figures of CNMs and DNB's validation on iEEG

Here, we list all the results on the early-warning signal of iEEG through CNMs and DNB. DNB fails in several cases because DG only has a single element clustered by the K-means algorithm, thus PCC_d is not well-defined this time.

ID1Sz1

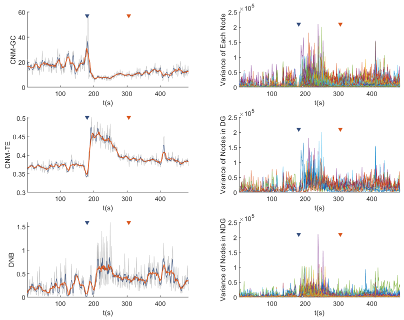

ID1Sz2

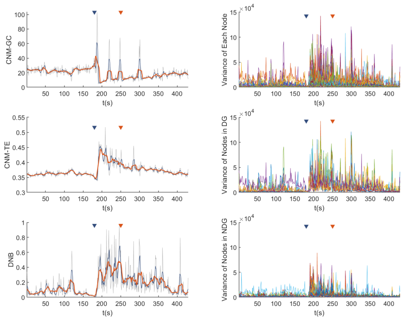

ID1Sz3

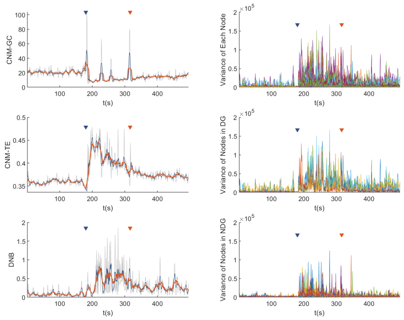

ID1Sz4

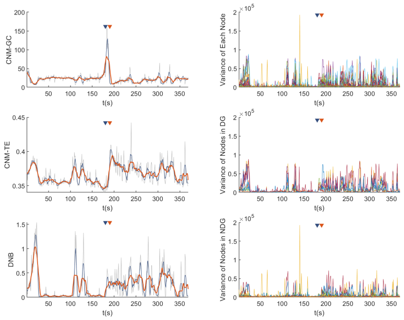

ID1Sz5

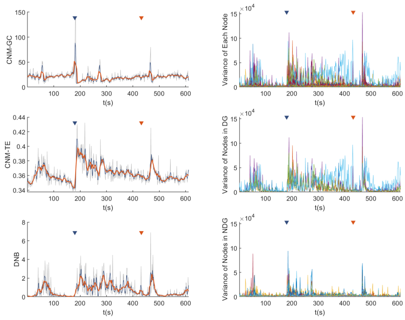

ID1Sz6

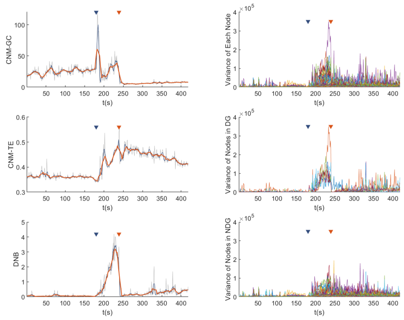

ID1Sz7

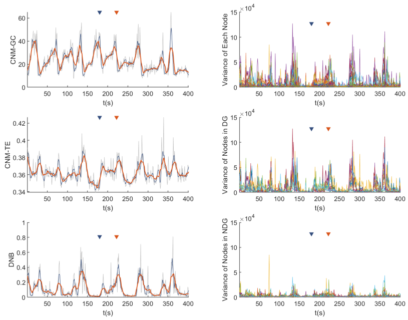

ID1Sz8

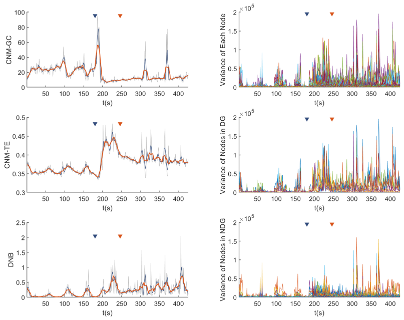

ID1Sz9

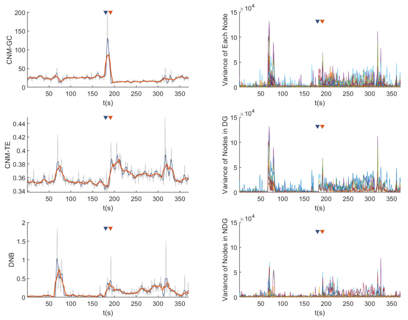

ID1Sz10

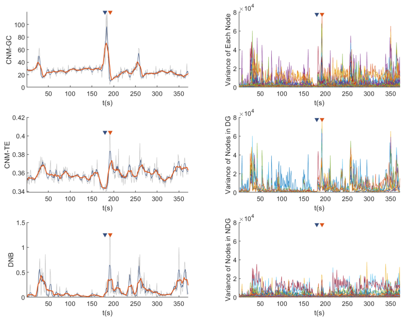

ID1Sz11

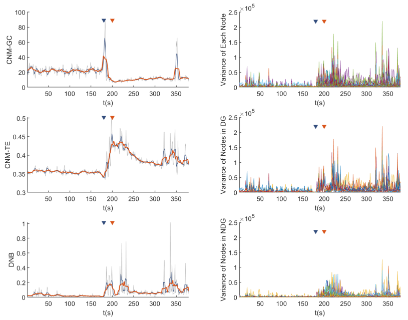

ID1Sz12

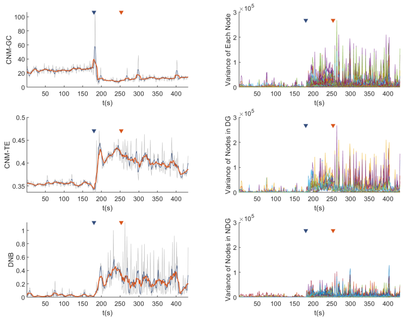

ID1Sz13

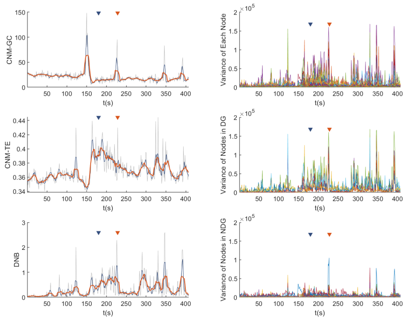

ID2Sz1

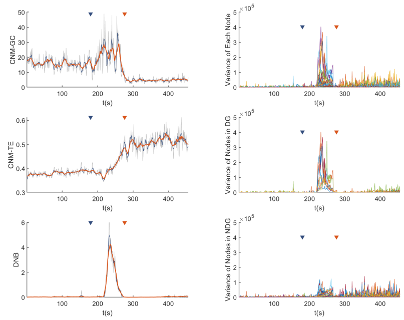

ID2Sz2

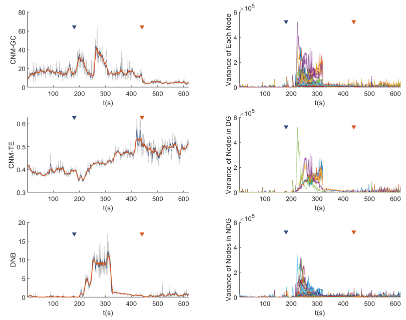

ID2Sz3

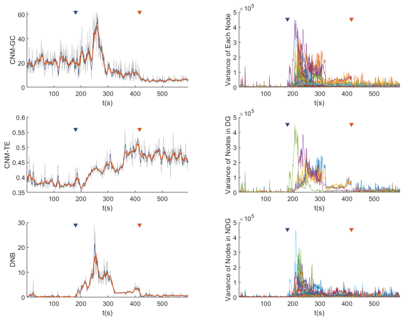

ID2Sz4

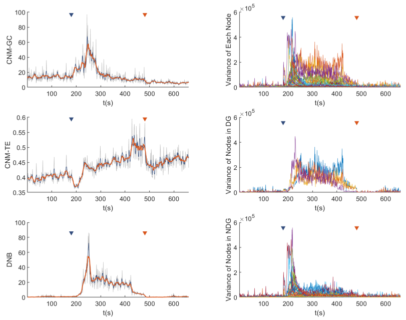

ID3Sz1

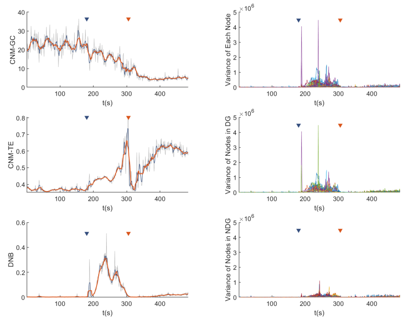

ID3Sz2

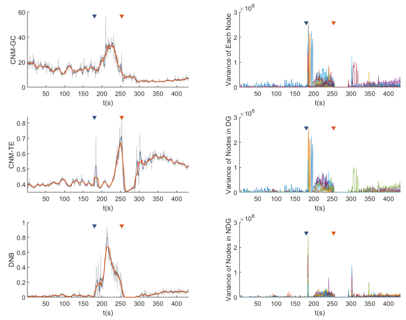

ID4Sz1

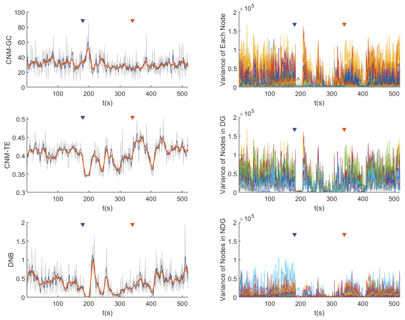

ID4Sz2

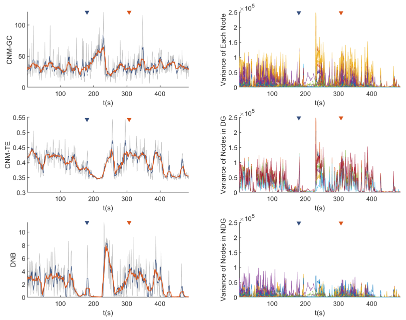

ID4Sz3

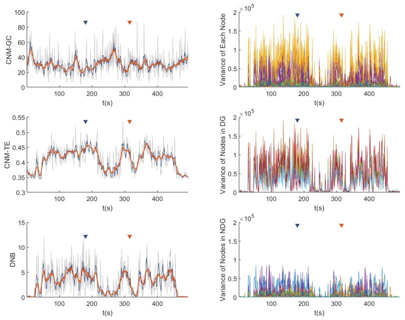

ID4Sz4

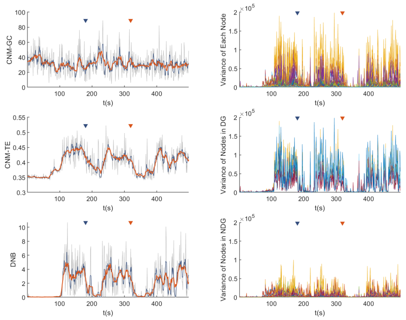

ID4Sz5

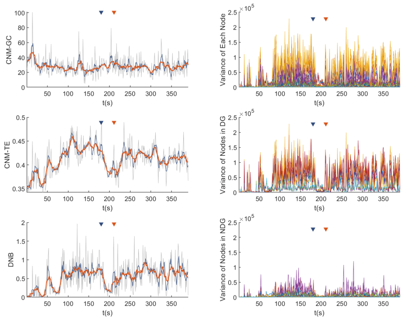

ID4Sz6

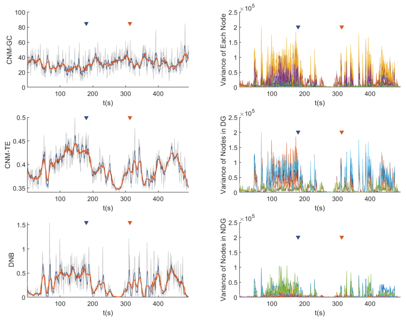

ID4Sz7

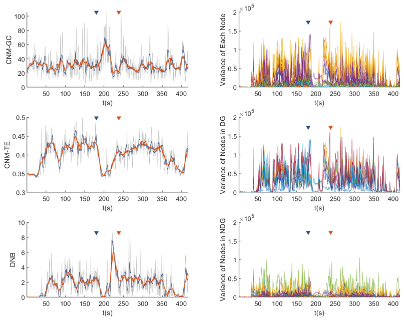

ID4Sz8

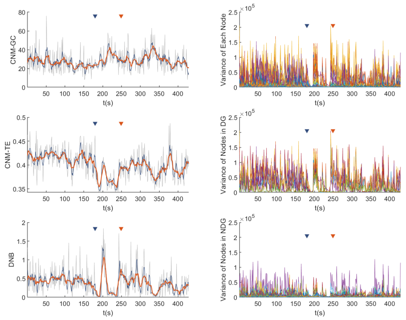

ID4Sz9

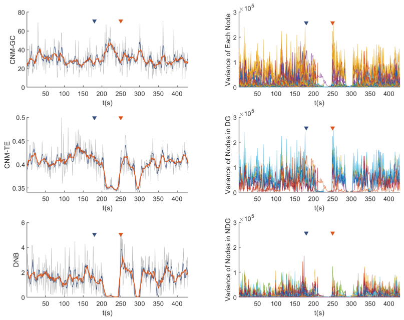

ID4Sz10

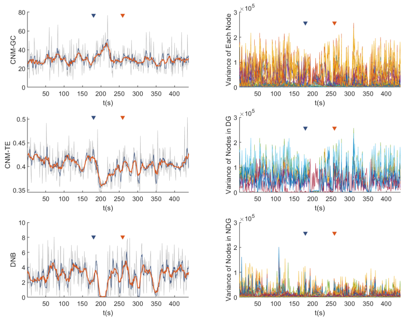

ID4Sz11

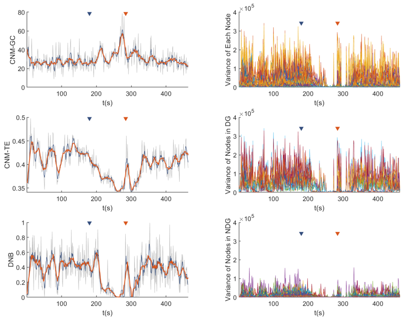

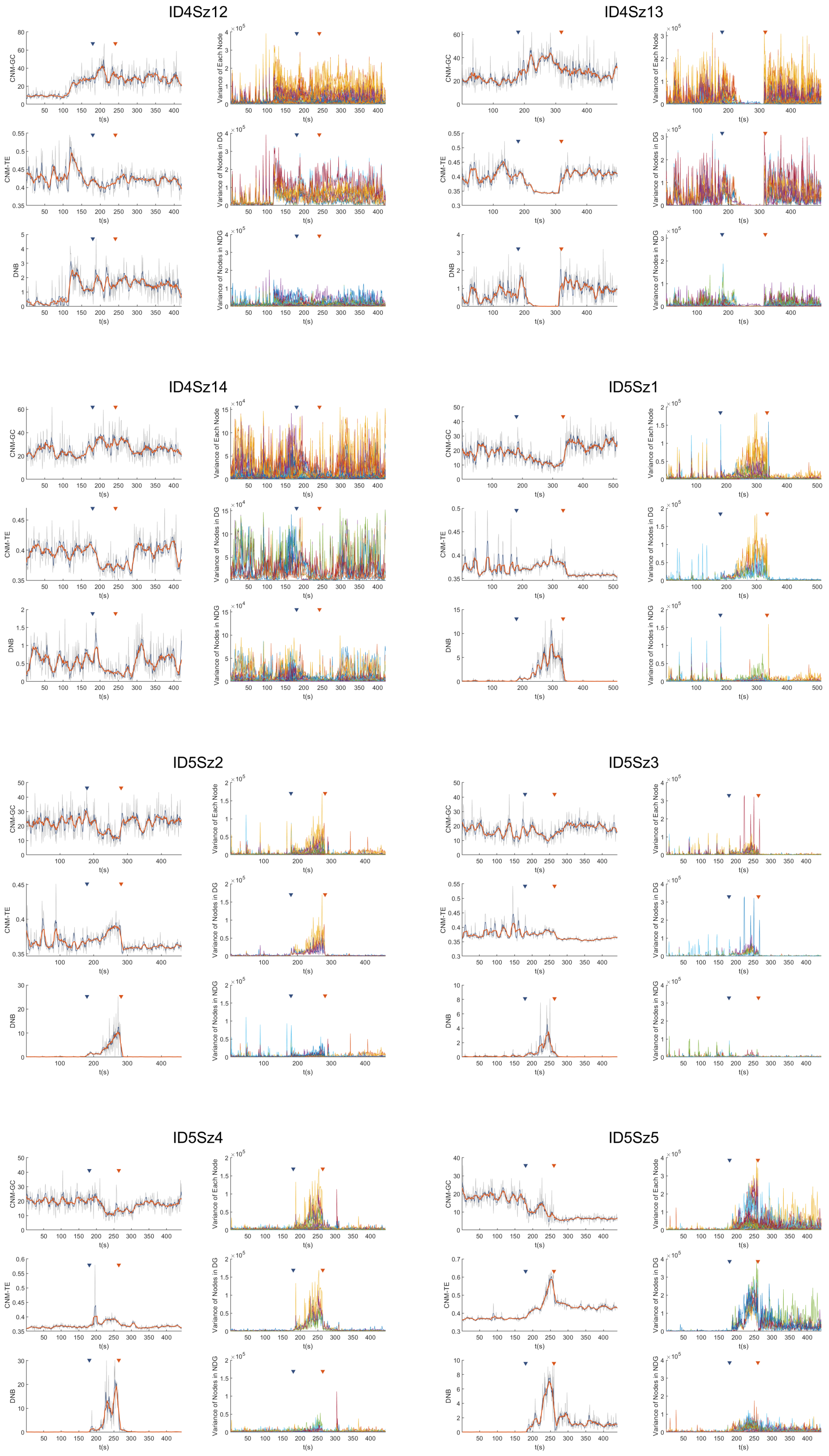

ID4Sz12
ID4Sz13
ID4Sz14
ID5Sz1
ID5Sz2
ID5Sz3
ID5Sz4
ID5Sz5
CNM-GC
CNM-TE
DNB
Variance of Each Node
Variance of Nodes in DG
Variance of Nodes in NDG
t(s)

ID5Sz6

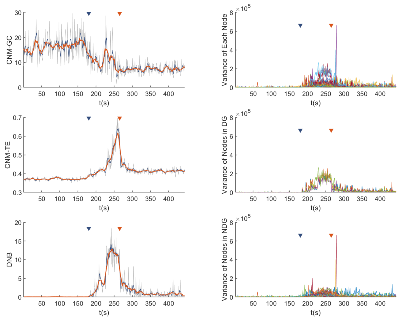

ID5Sz7

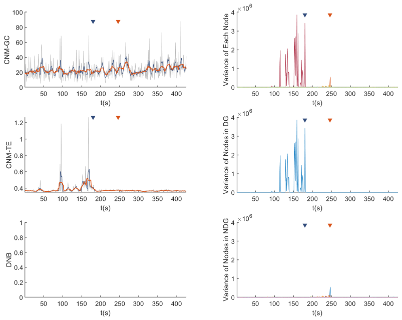

ID5Sz8

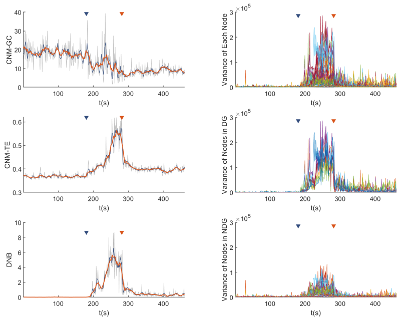

ID5Sz9

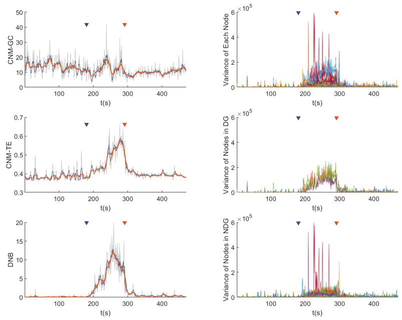

ID5Sz10

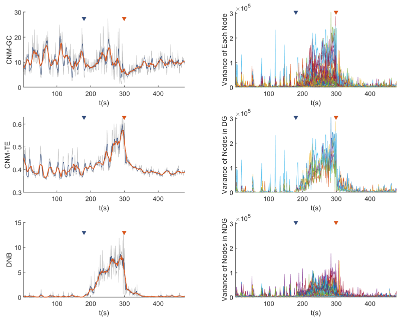

ID6Sz1

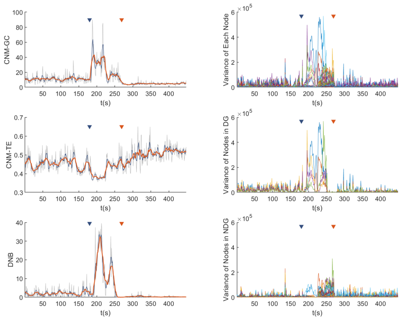

ID6Sz2

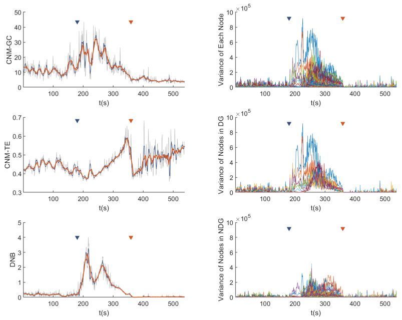

ID6Sz3

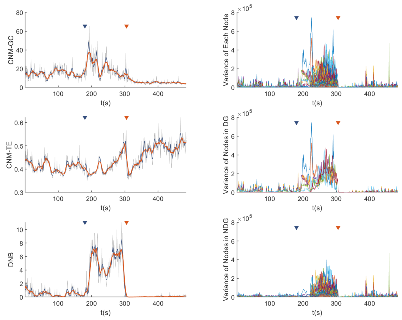

ID6Sz4

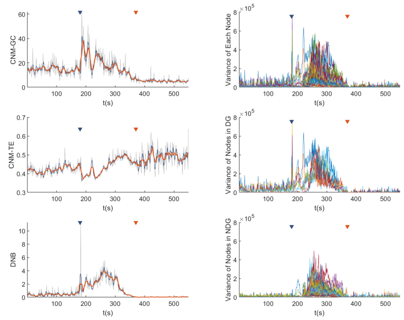

ID7Sz1

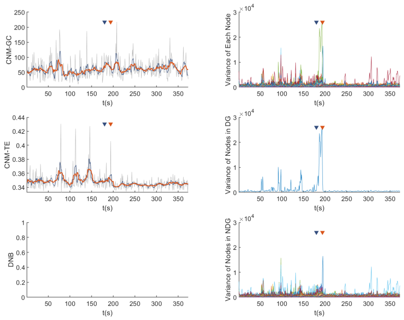

ID7Sz2

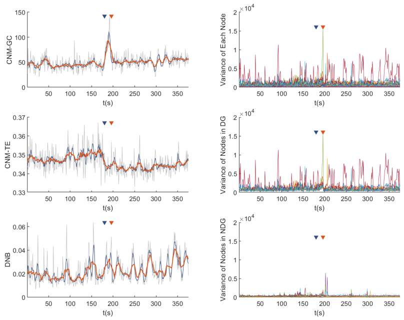

ID8Sz1

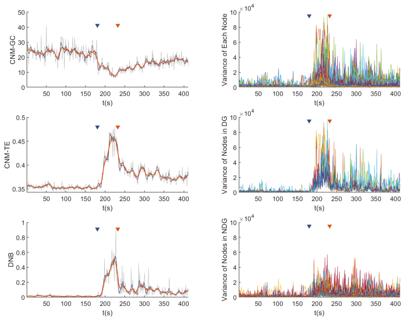

ID8Sz2

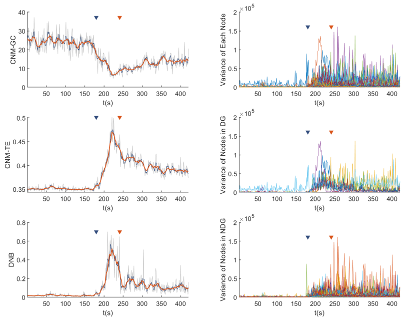

ID9Sz1

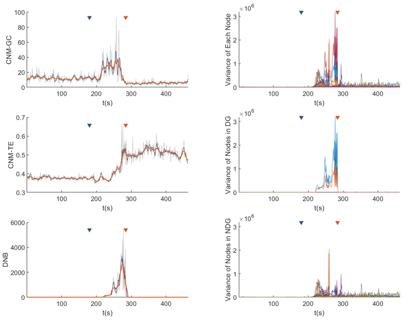

ID9Sz2

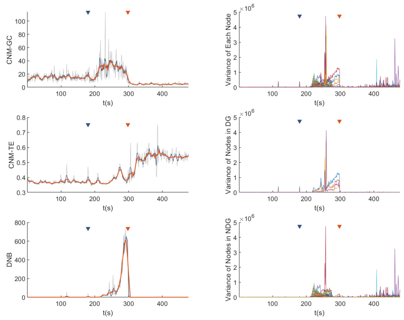

ID9Sz3

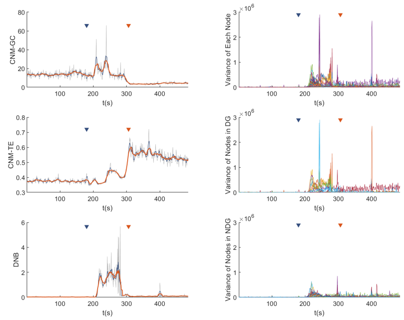

ID9Sz4

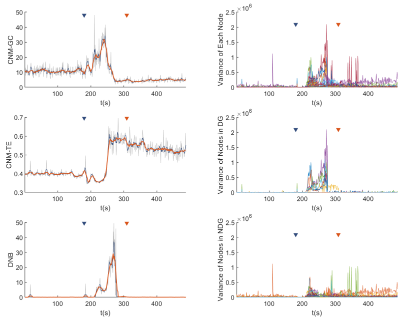

ID9Sz5

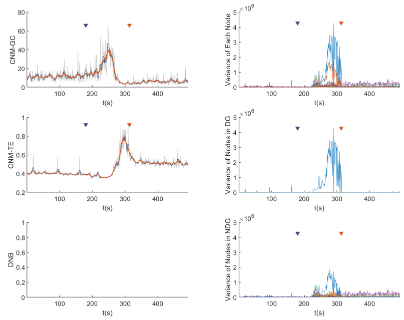

ID9Sz6

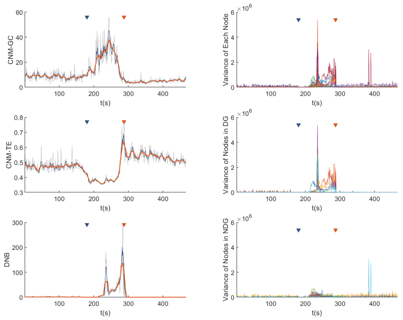

ID9Sz7

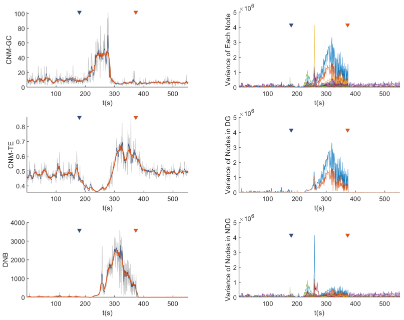

ID9Sz8

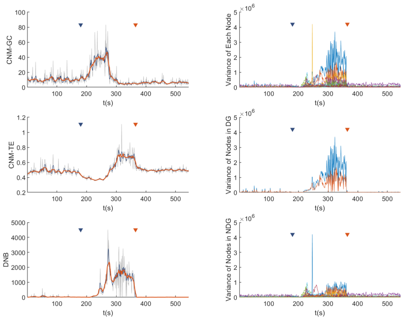

ID9Sz9

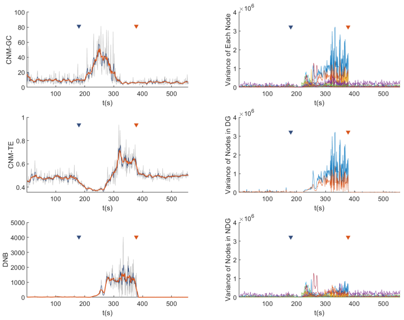

ID10Sz1

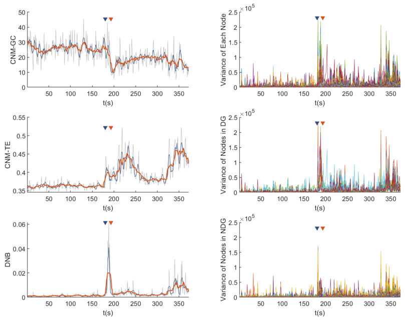

ID10Sz2

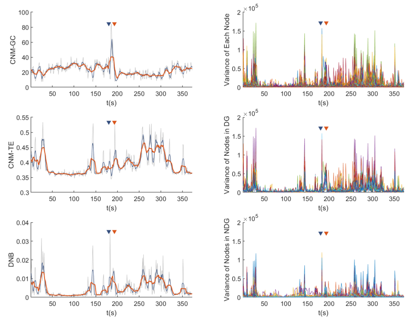

ID10Sz3

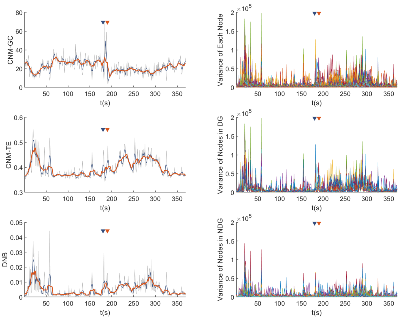

ID10Sz4

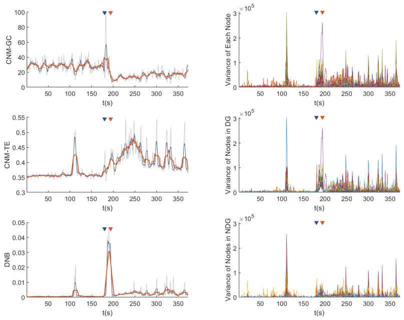

ID10Sz5

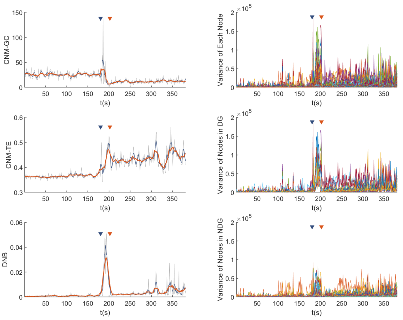

ID11Sz1

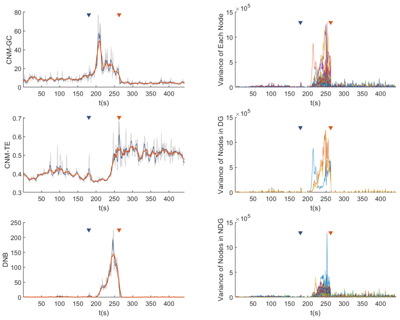

ID11Sz2

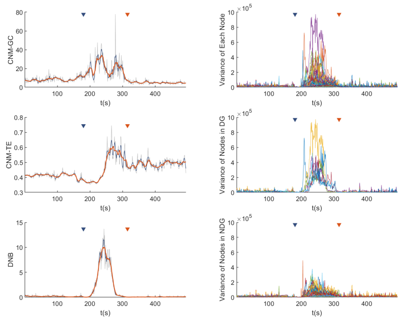

ID12Sz1

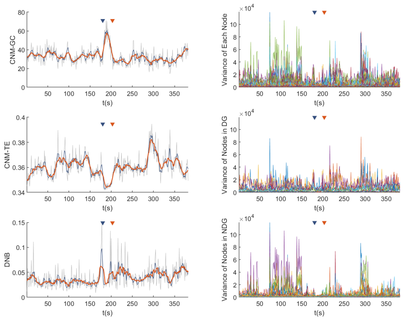

ID12Sz2

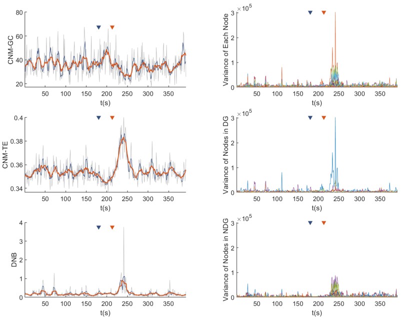

ID12Sz3

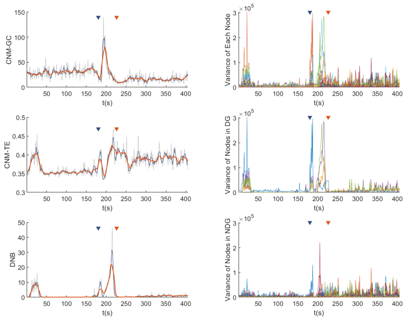

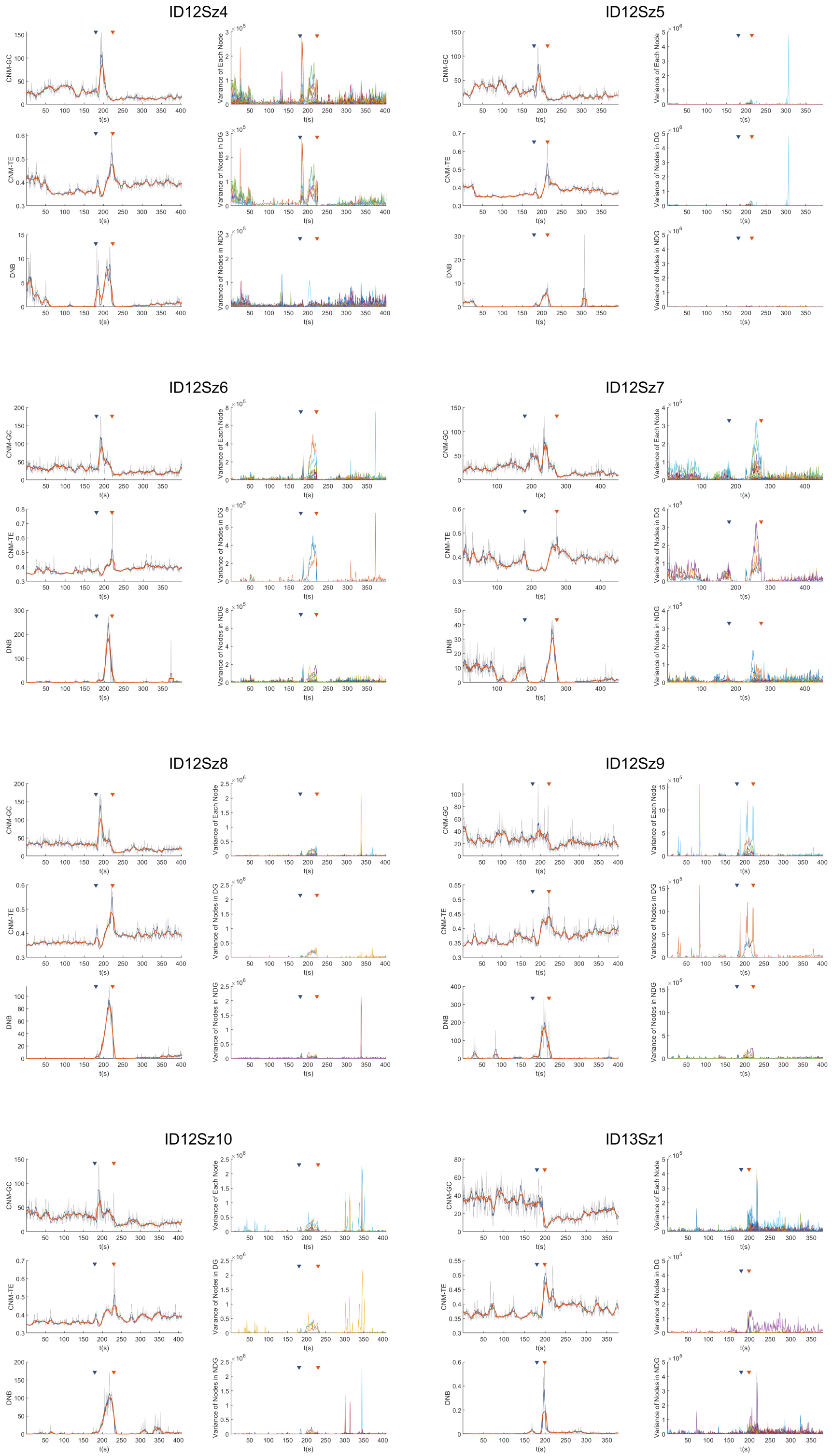
ID12Sz4
ID12Sz5
ID12Sz6
ID12Sz7
ID12Sz8
ID12Sz9
ID12Sz10
ID13Sz1
CNM-GC
CNM-TE
DNB
Variance of Each Node
Variance of Nodes in DG
Variance of Nodes in NDG
t(s)

ID13Sz2

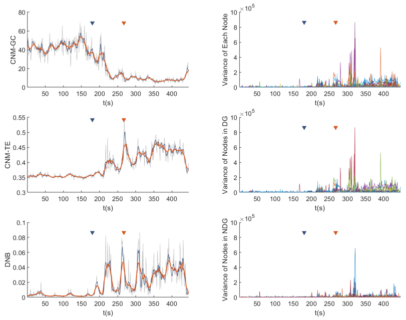

ID13Sz3

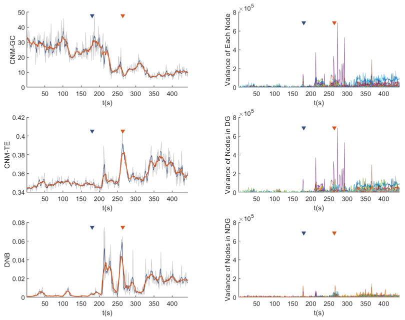

ID13Sz4

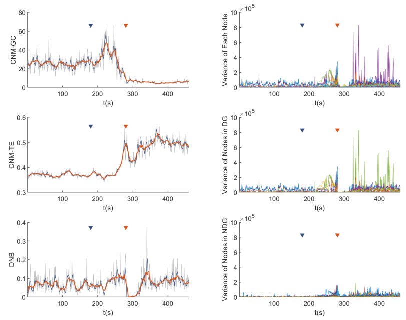

ID13Sz5

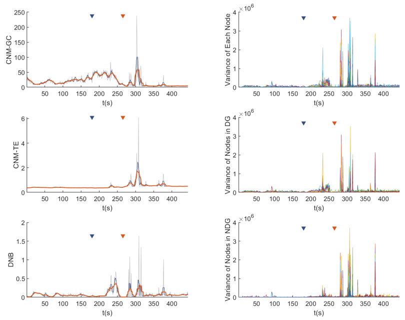

ID13Sz6

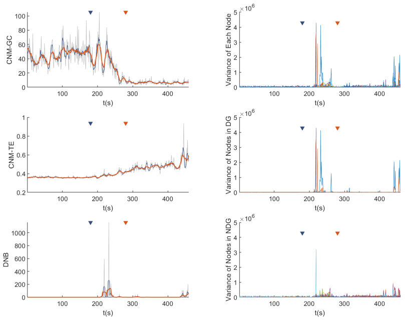

ID13Sz7

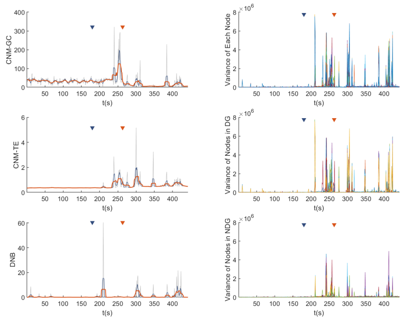

ID14Sz1

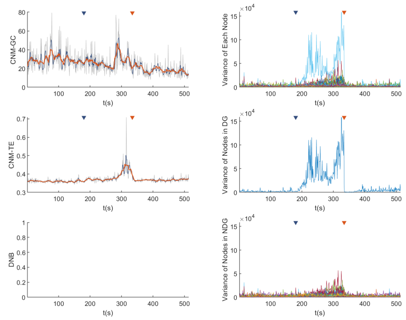

ID14Sz2

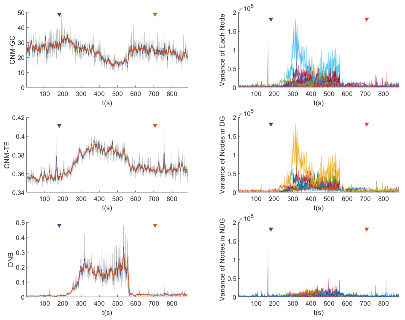

ID14Sz3

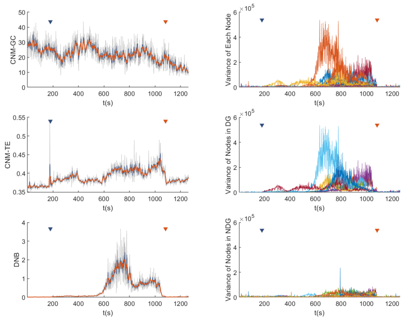

ID14Sz4

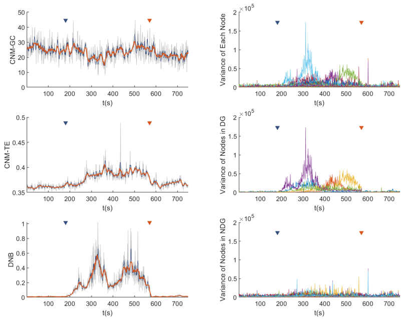

ID14Sz5

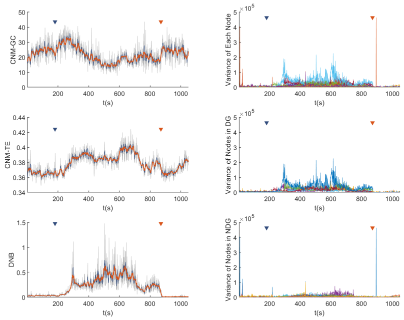

ID14Sz6

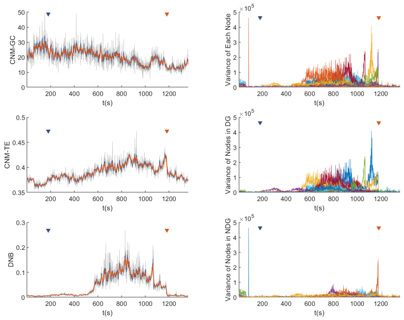

ID14Sz7

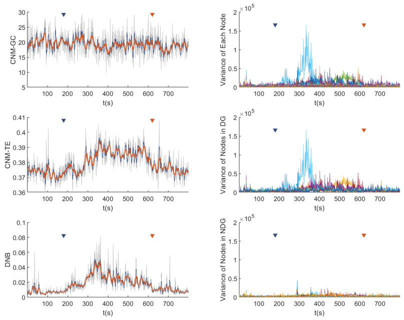

ID15Sz1

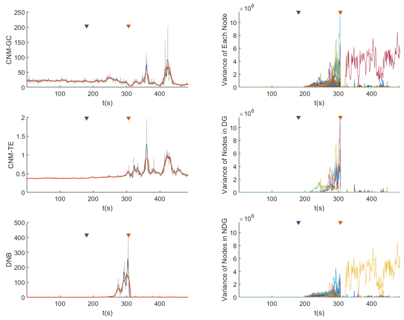

ID15Sz2

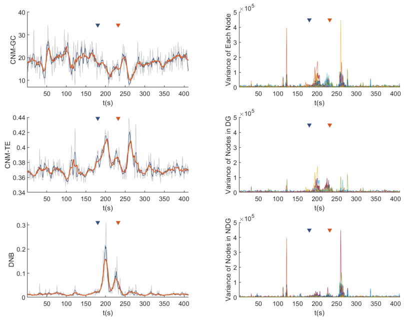

ID15Sz3

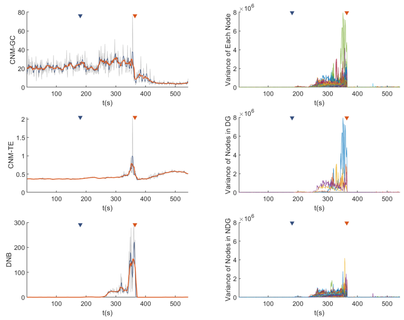

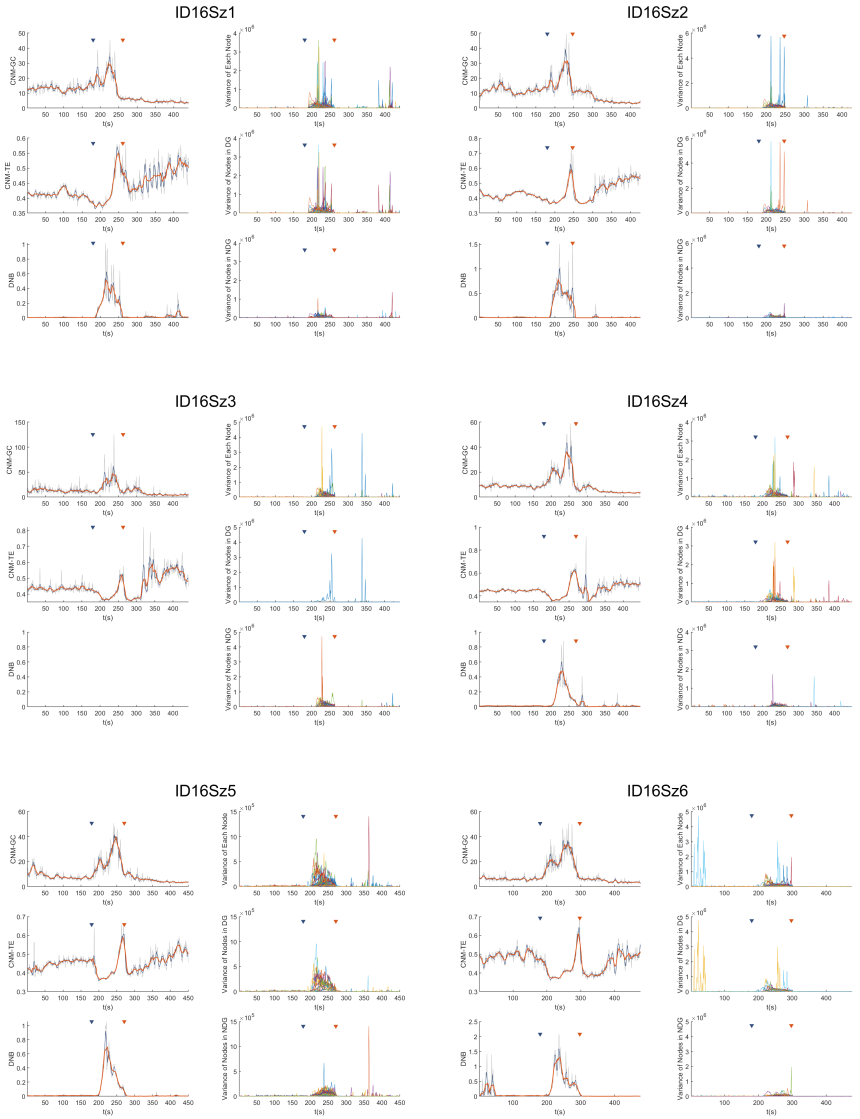

Code availability

The CNMs implementation code is available on GitHub: https://github.com/wzzzzzyb/CNMs.

References

[1] J. Shi, L. Chen, K. Aihara, Embedding entropy: a nonlinear measure of dynamical causality. Journal of The Royal Society Interface **19**(188), 20210,766 (2022)

[2] C. Ito, H. Takahashi, M. Ohzono, Estimation of convergence boundary location and velocity between tectonic plates in northern hokkaido inferred by gnss velocity data. Earth, Planets and Space **71**(1), 86 (2019). https://doi.org/10.1186/s40623-019-1065-z

[3] Y. Tong, R. Hong, Z. Zhang, K. Aihara, P. Chen, R. Liu, L. Chen, Earthquake alerting based on spatial geodetic data by spatiotemporal information transformation learning. Proc. Natl. Acad. Sci. U. S. A. **120**(37), e2302275,120 (2023)

[4] J. Parvizi, S. Kastner, Promises and limitations of human intracranial electroencephalography. Nature Neuroscience **21**, 474–483 (2018). https://doi.org/10.1038/s41593-018-0108-2

[5] A. Burrello, K. Schindler, L. Benini, A. Rahimi, in *Proceedings of the IEEE Biomedical Circuits and Systems Conference (BioCAS)* (IEEE, 2018)

[6] A. Burrello, K. Schindler, L. Benini, A. Rahimi, Hyperdimensional computing with local binary patterns: One-shot learning of seizure onset and identification of ictogenic brain regions using short-time ieeg recordings. IEEE Transactions on Biomedical Engineering (2019)

[7] M.I. Maturana, C. Meisel, K. Dell, P.J. Karoly, W. DSouza, D.B. Grayden, A.N. Burkitt, P. Jiruska, J. Kudlacek, J. Hlinka, M.J. Cook, L. Kuhlmann, D.R. Freestone, Critical slowing down as a biomarker for seizure susceptibility. Nature Communications **11**(1), 2172 (2020). https://doi.org/10.1038/s41467-020-15908-3. URL https://doi.org/10.1038/s41467-020-15908-3. PMID: 32437426
